# Abstract rule generalization for composing novel meaning recruits a frontoparietal control network

**DOI:** 10.1162/IMAG.a.963

**Published:** 2025-10-29

**Authors:** Xiaochen Y. Zheng, Mona M. Garvert, Hanneke E. M. den Ouden, Lisa I. Horstman, David Richter, Roshan Cools

**Affiliations:** Donders Center for Cognitive Neuroimaging, Radboud University, Nijmegen, the Netherlands; Cognitive Psychology Unit, Institute of Psychology, Leiden University, Leiden, the Netherlands; Julius-Maximilians-Universität Würzburg, Faculty of Human Sciences, Würzburg, Germany; Donders Center for Cognition, Radboud University, Nijmegen, the Netherlands; Radboud University Medical Center, Department of Psychiatry, Nijmegen, the Netherlands; Mind, Brain and Behavior Research Center (CIMCYC), University of Granada, Granada, Spain

**Keywords:** abstract rule learning, compositional generalization, cognitive control, linguistic inference, representational similarity analysis, frontoparietal network

## Abstract

The ability to generalize previously learned knowledge to novel situations is crucial for adaptive behavior, representing a form of cognitive flexibility that is particularly relevant in language. Humans excel at combining linguistic building blocks to infer the meanings of novel compositional words, such as “un-reject-able-ish”. The neural mechanisms and representations required for this ability remain unclear. To unravel these, we trained participants on a semi-artificial language in which the meanings of compositional words could be derived from known stems and unknown affixes, using abstract relational structure rules (e.g., “good-kla” which means “bad”, where “-kla” reverses the meaning of the stem word “good”). According to these rules, word meaning depended on the sequential relation between the stem and the affix (i.e., pre- vs. post-stem). During fMRI, participants performed a semantic priming task, with novel compositional words as either sequential order congruent (e.g., “short-kla”) or incongruent primes (e.g., “kla-short”), and real words serving as targets that were synonyms of the composed meaning of the congruent primes (e.g., “long”). Our results show that the compositional process engaged a broad temporoparietal network, while representations of composed word meaning were localized in a more circumscribed left-lateralized language network. Strikingly, newly composed meanings were decodable already at the time of the prime in a way that could not be accounted for representations of the prime words themselves. Finally, we found that the composition process recruited abstract rule representations in a bilateral frontoparietal network, in contrast to our preregistered prediction of a medial prefrontal-hippocampal network. These results support the hypothesis that people activate a bilateral frontoparietal circuitry for compositional inference and generalization in language.

## 1 Introduction

The ability to generalize previously acquired information to novel scenarios is essential for adaptive behavior in a changing world. While this hallmark of human cognition underpins learning and problem-solving across various cognitive domains (Behrens et al., 2018; Dehaene et al., 2022; Frankland & Greene, 2020; Gärdenfors, 2004; Schwartenbeck et al., 2023), this capacity is particularly clearly illustrated by language. When encountering the novel word “un-reject-able-ish” for the first time, we can swiftly infer its meaning by generalizing from the known elements and integrating them according to abstract relational structure rules, such as the sequential arrangement of word parts (Tamminen et al., 2015; Zheng, Petukhova, et al., 2024). We excel at combining linguistic building blocks such as morphemes and words to form larger structures like phrases and sentences, thereby flexibly conveying an infinite array of thoughts and ideas. The generation of linguistic meaning relies not only on the constituent parts, but more importantly, also on the abstract relational structure rules based on which they are combined (Fodor, 1975; Fodor & Pylyshyn, 1988; Frege, 1892; Martin, 2016; Partee, 2008). Consider the sentences “The cat chased the mouse” and “The mouse chased the cat”. Despite sharing identical linguistic building blocks, they convey distinct meanings. What neural mechanisms enable us to infer novel compositional meaning based on such rules? Does our brain represent abstract rules to facilitate meaning generalization, and if so, which circuits are recruited?

In cognitive neuroscience, extensive research has been dedicated to understanding how our brain organizes knowledge to guide flexible behavior. An influential line of inquiry has focused on how this organization is achieved through learning simplified and abstract representations of the world, formatted as cognitive maps (Constantinescu et al., 2016; Moser et al., 2008; O’Keefe & Nadel, 1978; Solomon et al., 2019; Tolman, 1948; Zheng, Hebart, et al., 2024). These relational knowledge structures allow us to infer associations that have not been directly experienced, and to generalize those abstract structures to novel situations (Bein & Niv, 2023; H. Eichenbaum & Cohen, 2014; Piaget, 1929; Preston & Eichenbaum, 2013). In a recent study, Schwartenbeck et al., (2023) investigated the neural representations and mechanisms that enable compositional generalization in the domain of vision. Participants solved compositional problems by inferring the relational positions of building blocks in a visual silhouette (e.g., a building block on top of vs. below another building block). Using fMRI, they found generalizable, relational configurations of visual building blocks to be represented in a medial prefrontal-hippocampal network. The same network has been shown to be recruited during various other forms of generalization, ranging from discovering a shortcut in spatial navigation (Epstein et al., 2017; Jacobs et al., 2013; Moser et al., 2008; O’Keefe & Nadel, 1978; Tolman, 1948), to “joining the dots” between events (Barron et al., 2013, 2020; Garvert et al., 2023; Morton et al., 2020), and to inferring unknown relationships in social contexts (Park et al., 2020, 2021). It has been proposed that neural cognitive map-like representations in circuitry connecting the hippocampus with the medial frontal cortex can serve as a universal knowledge code for generalization and novel inference across multiple cognitive domains (Behrens et al., 2018; Bellmund et al., 2018; Stachenfeld et al., 2017; Whittington et al., 2018).

In language sciences, the investigation of compositional generalization has, however, primarily implicated neural networks other than this medial prefrontal-hippocampal network. Compositionality—the ability to combine lexical building blocks to create linguistic meaning (Fedorenko et al., 2016; Gwilliams, 2020; Hagoort, 2019a, 2019b; Hagoort & Indefrey, 2014; Martin, 2020; Pylkkänen, 2019; Zaccarella et al., 2017; Zaccarella & Friederici, 2015)—is thought to rely on left-lateralized, language-specific networks, particularly in regions such as the left inferior frontal gyrus (Bozic et al., 2007; Bozic & Marslen-Wilson, 2010; Hagoort, 2005, 2016; Leminen et al., 2019; Nevat et al., 2017) and the left anterior temporal lobe (Baron & Osherson, 2011; Brennan et al., 2012; Flick et al., 2018; Pylkkänen, 2019). This suggests that compositional inference in language might engage neural systems distinct from those involved in compositional processes in relational memory, action planning and vision, challenging the notion that hippocampal-based representational codes are domain-general.

In the current preregistered fMRI study, we aimed to test this hypothesis by investigating the neural mechanisms underlying the ability to infer novel compositional word meanings based on abstract relational structure rules. Specifically, we aimed to assess whether relational structure-based composition in language recruits the medial prefrontal-hippocampal network that has also been implicated in action planning, visual composition, and relational memory (Baram et al., 2021; Barron et al., 2020; Schwartenbeck et al., 2023). To this end, we employed a recently developed language-learning paradigm where participants generalize abstract rules to infer novel compositional meanings (Zheng, Petukhova, et al., 2024). Unlike existing procedures that decode neural representations during natural language comprehension (e.g., Huth et al., 2016), this controlled experimental paradigm uses a semi-artificial language to isolate generalizable abstract rules for meaning composition and to probe core cognitive mechanisms that are otherwise difficult to disentangle in natural language. In this task, participants infer abstract rules from linguistic exemplars, then use these rules to derive the meanings of novel compositional words. According to these rules, word meaning depends on the sequential relation between the stem and the affix (i.e., pre- vs. post-stem). The paradigm was designed to capture (i) the observation that sequential order plays a key role in compositionality in natural language (Beyersmann & Grainger, 2023; Crepaldi et al., 2013, 2016), but furthermore, also (ii) the relational structure-dependent nature of compositional generalization in non-linguistic domains associated with hippocampal-medial frontal cortical circuitry (Baram et al., 2024; Barron et al., 2020; Garvert et al., 2023; Morton et al., 2020; Park et al., 2021; Schwartenbeck et al., 2023).

## 2 Methods

The study was approved by the local ethics committee (METC Oost-Nederland, 2014/288) and conducted in accordance with the Declaration of Helsinki. All participants provided written informed consent and received monetary compensation. The study was preregistered at AsPredicted (https://aspredicted.org/mk5i2.pdf).

### 2.1 Participants

Given the lack of prior data for this novel fMRI paradigm, we conducted a prior power analysis assuming a medium effect size (Cohen’s d = 0.5). This yielded a target sample size of 34 participants to achieve 80% power at an alpha of 0.05. To ensure that we retain sufficient data after applying standard MRI quality control and behavioral exclusion criteria, we planned a overshoot in recruitment. Specifically, we collected data from 43 right-handed, healthy Dutch native speakers (Mean_age_ = 23.1, SD_age_ = 4.3, range 18–33, 27 women, 15 men, 1 other). All participants had normal or corrected-to-normal vision. No participants reported any current or previous psychiatric or neurological disorders, nor MRI contraindications, such as unremovable metal parts in the body and claustrophobia. Seven participants were excluded due to various reasons, including scanner failure (N = 1), poor fMRI data quality (N = 3, see criteria in *MRI data acquisition and preprocessing*), falling asleep in the scanner (N = 1), or failure to learn to generalize the abstract rules (N = 4; 2 of which overlap with the ones with poor fMRI data, see criteria in *Behavioral analysis*), resulting in a dataset of 36 participants. In addition, 6 participants were excluded due to an unexpected error in the stimulus list in the scanning session. This left us with a final sample of 30 participants (Mean_age_ = 23.0, SD_age_ = 3.5, range = 18–30, 19 women, 11 men), slightly smaller than the planned target (N = 34).

### 2.2 Experimental paradigm

**Fig. 1.**
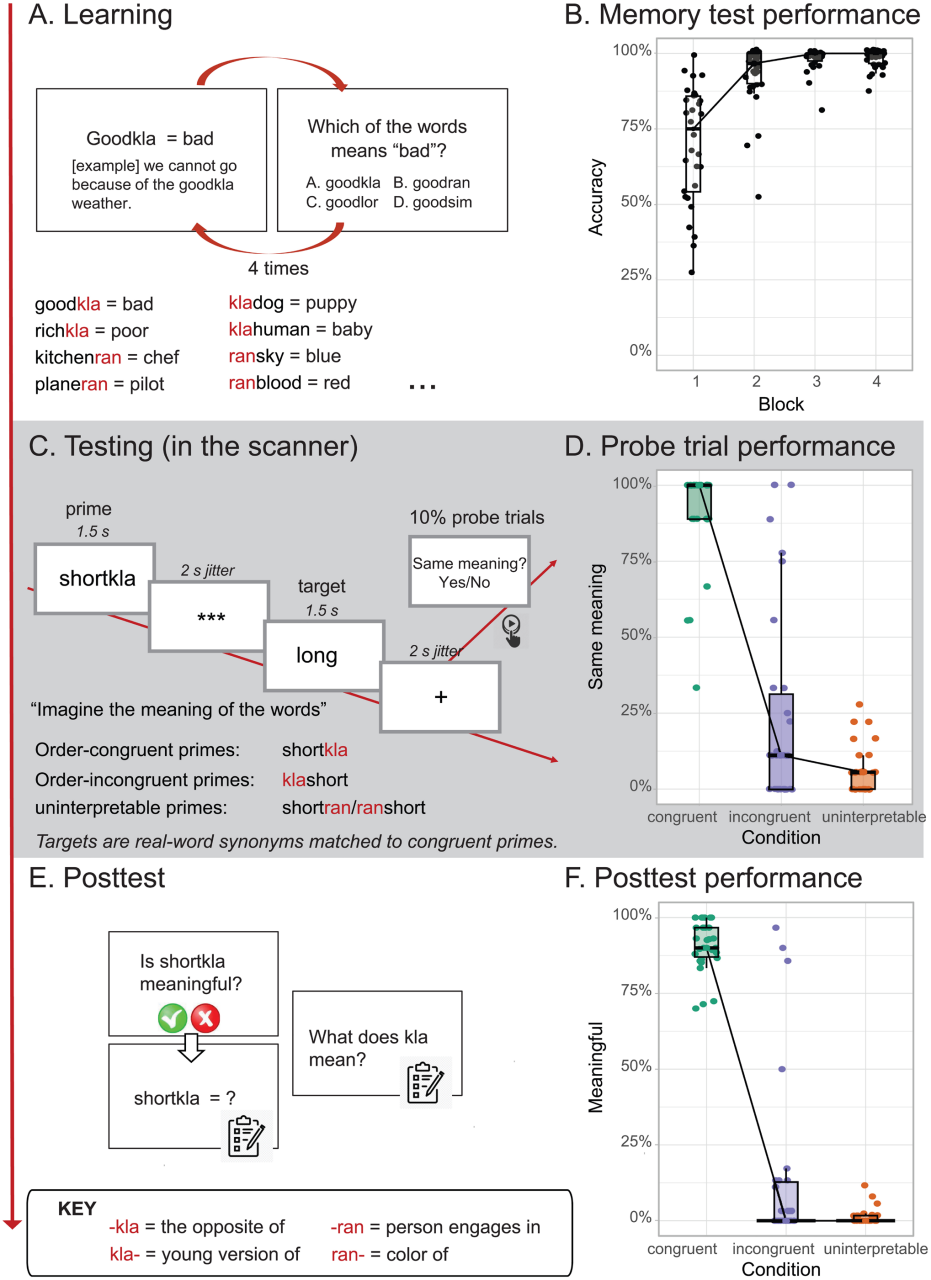
Experimental design (A, C, E) and behavioral results (B, D, F). (A) Participants learned and memorized artificial, compositional words. These compositional pseudo-words consisted of a known stem and an unknown affix. The affix alters the word meaning depending on its position (pre- vs. post- stem). Importantly, the abstract relational structure rules were never made explicit to the participants. (B) Box plots of participant’s choice performance in a memory task, where they recalled the meaning of the learned pseudo-words. (C) We tested participants’ knowledge with novel, compositional pseudo-words using an fMRI adaptation paradigm, in which the prime pseudo-words were always followed by a target, real word. The pseudo-word primes were either congruent or incongruent with the sequential order specified by the abstract rules, or belonged to a third condition in which the primes lacked interpretable meaning, regardless of order. The target word was always a matched synonym to the congruent prime word. (D) Boxplots of participants’ responses in the fMRI task across three experimental conditions, on the 10% probe trials on which they indicated with a left or right button press whether the prime words did or did not match the target words in terms of their meaning. (E) After the fMRI session, we explicitly asked participants to evaluate the meaningfulness of the novel compositional words. (F) Boxplots of participants’ responses in the posttest across three experimental conditions, where they indicated whether the pseudo-words are meaningful or not. The actual stimuli used in the experiment were in participants’ native language, Dutch (Supplementary Material 1). For (B, D, F), the thick horizontal line inside the box indicates the group median, and the bottom and top of the box indicate the group-level first and third quartiles of each condition. Each dot represents one participant. The black lines connect the group median across conditions.

To quantify participants’ ability to construct compositional word meaning by generalizing abstract relational structure rules, we employed an experimental paradigm where participants learned a semi-artificial language featuring various rules of compositions (Zheng, Petukhova, et al., 2024). A schematic diagram of the experiment is provided in Figure 1.

#### 2.2.1 Design

During a pre-scanning training phase, participants were exposed to pairs of compositional pseudo-words along with their experimentally assigned meanings (Fig. 1A, Supplemental Material 1). Each of these compositional pseudo-words comprised a known stem (e.g., “good” in “good-kla”) and an unknown affix (e.g., “kla”). Going beyond previous work on linguistic generalization (e.g., Tamminen et al., 2015), we designed the experiment such that meaning inference required the processing of the relational structure of the pseudo-word. Specifically, we manipulated the mapping of the meaning to the affix based on its sequential position: e.g., “-kla” as a suffix meant “the opposite”, whereas “kla-” as a prefix meant “young version”. These position-dependent rules allowed participants to compose unique meanings based on different sequential combinations of the affixes with the stems. Crucially, while the participants could infer the rules from the exemplars, these rules were never made explicit to them.

To test participants’ knowledge of abstract relational structure rules, after training, we presented a new set of compositional pseudo-words which they had never encountered before (e.g. “short-kla” and “kla-short”) and asked them to imagine the meanings of the words while recording fMRI. These novel pseudo-words were designed to either create conflict or not based on the application of the abstract rules regarding sequential order for meaning inference. For example, for “short-kla”, “-kla” as a suffix means “the opposite”, and the opposite of short is “long”. Conversely, for “kla-short”, “kla-” as a prefix means “the young version of”, and it is much more difficult to infer the meaning of the young version of “short”. These two types of pseudo-words—order-congruent and order-incongruent—were presented as primes and paired with real-word targets that were always synonyms of the congruent meaning (e.g., “short-kla” or “kla-short” followed by “long”; Fig. 1C).

We further included a third condition of pseudo-words, where the stems were combined with alternative affixes in such a way that the combination yielded uninterpretable meanings regardless of the position of the affix (e.g., ran-short = the color of short; short-ran = the person who engages with short). As a result, these compositional words did not correspond to the target word meanings (e.g., ≠ long). Note that while a given affix was paired congruently with several different stems, each stem was congruently paired with only a single affix (and also appears in various control conditions).

The setup was optimized for capturing neural adaptation in fMRI and allowed us to assess activity in neural circuits commonly associated with novel inference and abstract rule-based generalization.

#### 2.2.2 Procedure

Both the pre-scanning training and posttest were carried out in a sound-proof testing booth adjacent to the MRI room. The experiment was run using the software Presentation (Version 20.2, Neurobehavioural System Inc, Berkeley, U.S.).

##### 2.2.2.1 Pre-scanning training

Participants studied the training set of 30 compositional pseudo-words in a self-paced manner. Every compositional word was presented together with its synonym meaning and an example sentence using the word in context, till a maximum of 15 s or participants pressing to continue. After viewing all the words, participants completed a multiple-choice test where on each trial, they were given a synonym meaning and asked to choose a matched compositional word. Each compositional word was presented once in a learning block and once in a memory test. All the words were presented in a pseudorandom order, with the same affix form or affix position repeated on no more than three consecutive trials. The learning blocks and memory tests were interleaved and repeated for four times, with 30 trials per block.

##### 2.2.2.2 Scanning session

Next, participants went through a testing session in the MRI scanner, where they were presented with the testing set of novel (i.e., never previously seen) compositional pseudo-words (*primes*), paired with real-word *targets* that were either matched or unmatched synonyms. Participants were asked to imagine the meaning of the words presented on the screen.

Each prime word was presented on the screen for 1500 ms, followed by a jittered screen of “***”. The target word was then presented on the screen for 1500 ms, followed by another jittered screen of a fixation. Then, the next trial started. Both jittered intervals were generated from a truncated exponential distribution with a mean of 2 s (range = 1.5 - 5 s). All prime words and target words were presented in black, in the center of a white screen. Each block started with a 2 s fixation. All the pairs of prime and target were presented in a pseudorandom order, with the following requirements: (1) the same affix form or affix position repeated on maximally three consecutive trials; (2) the same condition repeated maximally for three consecutive trials; (3) The same stem repeated at least five trials apart.

To ensure that participants paid attention to the prime words, we included probe questions on 10% of the trials where participants needed to indicate if the prime pseudo-word shared the same meaning as the target word (“probe trials”). They responded by pressing the left (“yes”) or the right button (“no”) on the button box using their right index finger or middle finger, respectively. The probe questions stayed on the screen for a maximum of 10 s or until participants responded. The task then proceeded with a jittered fixation followed by the next trial.

The task consisted of three blocks in total, with each prime-target pair in each condition presented once in each block. Prior to going into the scanner, participants went through a practice block in the behavioral booth, where they were familiarized with the task and received feedback on their performance.

##### 2.2.2.3 Posttest

In a post-test, participants were asked whether the compositional pseudo-words they had seen in the scanning session were meaningful, and if so, what they meant.

The whole session took about 3 h.

### 2.3 MRI data acquisition and preprocessing

#### 2.3.1 Data acquisition

The MRI experiment was performed on the institute’s 3T MAGNETOM Prisma[Fit] MR scanner (Siemens AG, Healthcare Sector, Erlangen, Germany) using a product 32-channel head coil. Out of the 30 participants in the final sample, 15 were scanned in a Prisma scanner and 15 were in a PrismaFit scanner. The assignments of participants were randomized. Despite the fact that the two scanners were theoretically the same, we additionally validated our results by including scanner as a second-level covariate. Our results were unchanged when adding scanner-type as covariance.

T2*-weighted blood-oxygen-level-dependent (BOLD) images were acquired in three blocks, recorded using a whole-brain multiband accelerated echo-planar imaging (EPI) sequence [TR, 1500 ms; TE, 39.6 ms; multiband acceleration factor, 4; flip angle, 75°; slice matrix size, 104 × 104; voxel size, 2.0 mm isotropic; FoV, 210 × 210 × 136 mm; bandwidth: 2090 Hz/px; echo spacing: 68 ms]. A high-resolution structural image (1 mm isotropic) was acquired using a T1-weighted 3D magnetization-prepared rapid gradient-echo sequence (MP-RAGE; TR, 2300 ms; TE, 3.03 ms; flip angle, 8°; FoV, 256 × 256 × 192 mm).

#### 2.3.2 MRI quality control

The MRI quality control was performed using MRIQC 22.0.6. (Esteban et al., 2017). Means of framewise displacement (both in mm and in percentage of timepoints), temporal SNR, and DVAR for functional images were computed per participant per block based on the image quality metrics. Blocks with any of these values larger than 2.5 SD from the group mean were excluded (or smaller than 2.5 SD for temporal SNR). Individuals with two or more blocks excluded were also excluded from the dataset (N = 3).

#### 2.3.3 Preprocessing

All MRI data were preprocessed using fMRIPrep 21.0.2 (Esteban, Blair, et al., 2018; Esteban, Markiewicz, et al., 2018; RRID:SCR_016216), which was based on Nipype 1.6.1 (Gorgolewski et al., 2011, 2018; RRID:SCR_002502). Information about the preprocessing of anatomical and functional data was retrieved directly from fMRIPrep and provided in Supplementary Material 2.

In addition, we used Statistical Parametric Mapping 12 (SPM12; Wellcome Trust Centre for Neuroimaging, https://www.fil.ion.ucl.ac.uk/spm/) to spatially smooth the final preprocessed BOLD time series with a 6 mm FWHM kernel.

### 2.4 Behavioral analysis

#### 2.4.1 Preprocessing

As a sanity check, we confirmed that all participants scored above chance-level (25%) in the memory test after the last block of learning and recalled more than half of the learned words in the posttest.

Participants’ written responses to the pseudo-word meaning in the posttest were coded as (1) matching the synonym, (2) meaningless, (3) creative, unexpected answers (e.g., when one consider a pseudo-word “human-kla” from the uninterpretable condition, the opposite of human, to be “animal”), and (4) unexpected but incorrect answers (e.g., when one confused the meaning of different affix forms, mistook “warm-ran” as “warm-kla‘ and reported the meaning to be “cold”, the opposite of warm). We excluded the unexpected cases from the analysis, which concerned 4.8% of the trials. Due to the paired presentations in the priming task (e.g., “short-kla” always followed by “long”), participants who judged a pseudo-word as meaningful in the post-test typically provided the target synonym as its inferred meaning (e.g., responding that “short-kla” meant “long”) in open-ended questions (Kendall’s τ = 0.98, *p* < .001). Therefore, we used the second measure—the percentage of inferred meaning matching the synonym word—as an indicator of participants’ explicit inference, as it offered greater certainty than a binary choice. Based on the post-test, we excluded participants who failed to learn the abstract rules (N = 4, of which 2 were the same participants excluded due to MRI quality control). They were defined as those who consider more than half of the pseudo-words in the uninterpretable condition to be meaningful, or more than half of the pseudo-words in the congruent condition to be meaningless.

#### 2.4.2 Statistical analyses

Behavioral data were submitted to generalized linear mixed models with the *glmmTMB* package (Version 1.9.11, Brooks et al., 2017) in R (Version 4.1.0; R Core Team, 2017). For the analysis of the probe trials and the post-test, we included experimental condition (order-congruent vs. order-incongruent vs. uninterpretable) as a predictor. Participants and items were included as random effects, with condition as a random slope for participants. The significance of condition was assessed using the Type II Wald Chi-square test. We used the *multcomp* package (Version 1.4.17, Hothorn et al., 2008) to conduct pairwise comparisons among the three experimental conditions.

### 2.5 fMRI analysis

fMRI data were analyzed using SPM12, the Matlab-based Representational similarity analysis (RSA) toolbox (Nili et al., 2014, https://github.com/rsagroup/rsatoolbox_matlab) and custom scripts written in MATLAB R2022b (Mathworks Inc.; https://nl.mathworks.com/products/matlab.html).

#### 2.5.1 Univariate analysis

To identify the neural BOLD signals associated with the compositional process, we compared fMRI responses during *order*-*congruent versus order-incongruent* primes, when participants first encountered the novel pseudo-words. To uncover the neural representations of the composed meanings, we exploited the phenomenon of fMRI adaptation (Barron et al., 2016; Grill-spector et al., 2006). This effect refers to a reduced neural response when the same neural population is repeatedly activated, with the degree of suppression scaling with the similarity between neural representations. Notably, suppression can also occur when different stimuli that share a relevant feature (e.g., semantic meanings) are presented in close succession (“cross-stimulus suppression”). Based on this principle, we reasoned that in brain regions involved in representing word meanings, neural response should be suppressed upon repeated exposure to the same semantic content—a well-established effect in semantic priming paradigms (Matsumoto et al., 2005; Wagner et al., 1997; Wible et al., 2006). In our task, neural signals at the time of the target would be suppressed to a greater degree when that target was preceded by a *order-congruent* prime word that shared the same meaning, compared with an ambiguous, *order-incongruent* prime with reversed structural order, reflecting the effect of abstract rules.

An event-related generalized linear model (GLM) was used to model both the prime and the target events, and contained separate onset regressors for each of the four experimental conditions (i.e., congruent, incongruent, and two times uninterpretable conditions—the latter counterbalanced to ensure the same amount of trials per affix type). The GLM also contained an onset regressor for the probe trials and a button press regressor as regressors of no interest. All regressors were convolved with a canonical hemodynamic response function. Because of the sensitivity of the blood oxygen level-dependent signal to motion and physiological noise, we included in the GLM the framewise displacement, six rigid-body motion parameters (three translations and three rotation), six anatomical component-based noise correction components (aCompCorr), and all the cosine regressors estimated by fmriprep as confound regressors for denoising. Each block was modeled separately within the GLM. The contrast images of all participants were then analyzed as a second-level random effects analysis.

Our preregistration included a planned contrast comparing the order-congruent/incongruent conditions to the uninterpretable condition for successful inference. However, to foreshadow the results, the participants did not distinguish between the order-incongruent and uninterpretable conditions in their behavioral responses. Given this observation, we focused the main fMRI analysis on the congruent and incongruent conditions. As a validation for successful vs. unsuccessful interference, analyses of prime- and target-related activities for the congruent versus uninterpretable contrast are provided in Supplementary Material 5C.

Based on previous work on nonlinguistic composition and generalization, particularly in the domain of relational memory (Barron et al., 2020; Garvert et al., 2023; Jacobs et al., 2013; Morton et al., 2020; Park et al., 2021; Schwartenbeck et al., 2023), we hypothesized that the process of composing novel meanings elicits activity in a circuit connecting the hippocampal formation with the medial prefrontal cortex. To test the engagement of this network, we conducted additional analyses using small volumes correction (SVC) within an anatomically defined ROI combining the hippocampal formation (incl. hippocampus, entorhinal cortex, subiculum) and a functionally defined medial prefrontal cortex (mPFC) ROI (Schwartenbeck et al., 2023). To examine the role of the language network in meaning inference, we performed additional SVC using two anatomically defined masks: the left inferior gyrus (IFG) and the left anterior temporal lobe (ATL). All ROIs are defined in Supplementary Materials 3. We considered our results significant if they survived family-wise error (FWE) correction at the cluster-level of *p* < .05 within these masks. Activations in other brain regions were only considered if they survived whole-brain cluster-level FWE correction at *p* < .05. All statistical parametric maps visualized in the manuscript were thresholded at *p* < .001 uncorrected and unmasked solely for illustration.

#### 2.5.2 Multivariate representational similarity analysis

To decode the neural representations of both the abstract relational structure rules and the newly inferred word meanings at the time of *prime*, we adopted a multivariate RSA approach (Kriegeskorte et al., 2008; Nili et al., 2014). Consider the compositional pseudo-word “short-kla”: To compose its meaning, participants would represent the *rule* (“-kla” means “the opposite of”); moreover, provided successful composition, they would also represent the composed *meaning* (“short-kla” means “long”). RSA allowed us to capture the relevant neural representations by computing the neural representational dissimilarity matrices (RDMs) based on prime-related fMRI activity for each pseudo-word, analyzed through a whole-brain searchlight. We assessed whether these neural RDMs were explained by model RDMs (see below) that capture the similarities between these pseudo-words as a function of either their composed meaning (derived from a word embedding model) or the rule that was used to compose them (by experimental design).

##### 2.5.2.1 Neural RDMs

To construct a neural RDM, we pairwise computed the similarities between multivariate neural activity patterns elicited by each pseudo-word primes and all others. Two primary types of neural RDMs were computed from the prime-related fMRI data: the first one irrespective of congruency, the second type modeling separately for the congruent and incongruent conditions. We expected both congruent and incongruent conditions to engage abstract rule representations, whereas only congruent primes to lead to target meaning representations.

For the first RDM, we estimated neural activity for each prime using a GLM that included separate onset regressors for each of the 30 compositional pseudo-words, collapsing across congruency (e.g., a single onset regressor for both “short-kla” and for “kla-short”). The resulting parameter estimates were used to compute a 30 × 30 neural RDM. For the second RDM, a separate GLM modeled the prime according to congruency (e.g., distinct onset regressors for “short-kla” and for “kla-short”). This produced a second type of neural RDMs reflecting condition-specific representations, including the congruent-only RDM and the incongruent-only RDM.

Both GLMs included additionally regressors of no interest: one for all prime trials in the uninterpretable condition, one for all the target words, one for the probe trials, and one for button presses. All regressors were convolved with a canonical haemodynamic response function. The same confound regressors as in the univariate analysis were included, and each block was modeled separately.

For rule representation, we began with the first neural RDM (i.e., including both congruent and incongruent primes), based on the expectation that both conditions engage abstract rule representations during the compositional process (i.e., at the time of the prime). Given the novel inference nature of the study, each pseudo-word was presented only once per block to avoid repetition. Collapsing across congruent and incongruent conditions also effectively doubled the number of trials per item across the three blocks. To further examine potential differences in rule representations between the congruent and incongruent conditions, we used the second set of condition-specific neural RDMs.

We reasoned that during incongruent primes, participants may not be representing the target word meanings. Therefore, we used the second, congruent-only RDM to assess meaning representations

In addition, we computed a third neural RDM using target-related fMRI data from all conditions (i.e., including the uninterpretable condition). This RDM served to validate the RSA procedure, specifically in relation to target word visual and meaning representations.

All RSA employed a whole-brain searchlight approach with a 7 mm spherical radius (approx. 180 voxels), with pairwise correlation distance (one minus Pearson correlation coefficient) as the distance metric.

##### 2.5.2.2 Model RDMs

We constructed two models of interest:

1. Meaning Model (Fig. 3A): This model captures representations of newly composed word meanings, arranged by their semantic similarities derived from a word embedding model (see below). For example, “long” (from “short-kla”) is more similar to “big” (from “small-kla”) than to “sad” (from “happy-kla”). We hypothesized that BOLD pattern similarity in brain regions encoding these newly constructed meanings (e.g., “short-kla” means “long”) should reflect the semantic similarity of the composed words (e.g., “long”).
2. Rule Model (Fig. 3B): This model captures the representation of abstract relational structure rules, where all compositional pseudo-words ending with “-kla” are more similar to each other than to pseudo-words with different affixes (e.g., “kla-”, “-ran”, or “ran-”). We expected that the BOLD patterns from brain regions encoding abstract rules would be best explained by this model.

The meaning model was constructed using embedding vectors for the 30 target words (e.g., “long” in “short-kla = long”) from a word embedding model (Mandera et al., 2017). Word embedding represent words in a continuous vector space, where similar meanings have similar representations. To limit the degrees of freedom in selecting from the many available language models, we opted for a relatively simple and well-established model—a Continuous Bag of Words (CBOW). This model has been shown to effectively predict human behavior of semantic priming in the Dutch-speaking population, consistent with our sample. Specifically, Mandera et al. (2017) evaluated several prediction-based language models against a large behavioral dataset. We used the best-fitting model there: CBOW model trained on the SONAR-500 text corpus (Oostdijk et al., 2013) and a corpus of movie subtitles. Pairwise Pearson correlation distances between target word embeddings formed a 30*30 distance matrix representing target meaning similarities. In addition, we constructed a stem meaning model based on the stems of the 30 prime words (e.g., “short” in “short-kla = long”).

For the rule model, we considered a 30*30 binary-coded distance matrix, where rules were either the same (e.g., “short-kla” and “happy-kla”) or different (“short-kla” and “kla-dog”).

As a sanity check for the RSA procedure, we computed two visual model RDMs to capture target word-related visual patterns and confirmed that the visual aspects of word forms were represented in the visual cortex (Supplementary Material 6A). Both RDMs reflect the visual similarity of target words presented on the screen: (1) Levenshtein distance, calculated using the “stringdist” library (van der Loo, 2014) in R; (2) Pixel-wise Euclidean distance between individual words. As expected, these two RDMs were highly correlated (Kendall’s τ = 0.57, *p* < .001).

##### 2.5.2.3 Statistics

Within each searchlight sphere for each participant, we compared the model RDMs with the neural RDMs using Kendall’s rank correlation. Both the searchlight analysis of the neural RDMs and the comparison with the model RDMs were conducted using the Matlab-based RSA toolbox (Nili et al., 2014). The resulting correlation coefficients were submitted to a one-sample t-test (i.e., contrasting the obtained correlation against zero) using SPM12. Statistical significance was assessed using cluster-inference with a cluster-defining threshold of *p* < .001 and whole-brain cluster-level FWE correction at *p* < .05.

Additionally, we conducted ROI-based RSA using the same hippocampal mask and the left IFG mask as in the univariate analysis. For each structural ROI, we followed the same procedure as the searchlight analysis, with first-level coefficients submitted to a group-level one-sample one-side t-test.

To estimate the explainable variance in the neural data—that is, the maximum correlation any model could reasonably achieve given the noise in the data—we calculated the lower bound of the noise ceiling using a leave-one-participant-out approach. For each participant, we correlated their neural RDM with the average neural RDM of all the other participants and then averaged these values across participants to obtain a conservative estimate of the noise ceiling. The noise ceiling was not used for statistical inference, but served as a descriptive benchmark to evaluate model performance.

## 3 Results

### 3.1 Generalization of abstract rules for novel meaning inference

The meanings of all pseudo-words were successfully learned during training, evidenced by ceiling level performance on a subsequent memory task that required recall of the meanings of these words (mean_accuracy_ = 98.2 %, SD = 3.1%, Fig. 1B).

To test participants’ knowledge of the abstract relational structure rules, we presented a new set of compositional pseudo-words that they had never encountered before (e.g. “short-kla” and “kla-short”) and asked them to imagine the meanings of the words, while recording fMRI. After 10% of the targets, participants were presented a probe question, asking whether the meaning of the target word was the same as that of the preceding, pseudo-word prime. Analysis of participants’ responses to these probe trials showed significantly higher probability of meaning-match responses in order-congruent (mean = 90.7%, SD = 16.5%) than incongruent trials (mean = 23.8%, SD = 32.3%; β = 4.52, SE = 0.78, z = 5.80, *p* < .001; Fig. 1D), evidencing their reliance on the abstract rules for inference. These results were validated in a posttest administered outside the scanner, where participants explicitly indicated whether they considered the novel pseudo-words that they had seen during the preceding MRI session to be meaningful or not (Fig. 1E, 1F, Supplementary Materials 4). Moreover, participants did not consider the uninterpretable pseudo-words to match the meaning of the real-word targets, and their responses in the uninterpretable condition did not differ from those in the incongruent condition (Supplementary Materials 4).

Together, these behavioral results demonstrate that participants were able to efficiently compute novel compositional meaning by generalizing previously learned abstract rules to new situations.

### 3.2 Compositional meaning representations in language-specific frontal regions

**Fig. 2.**
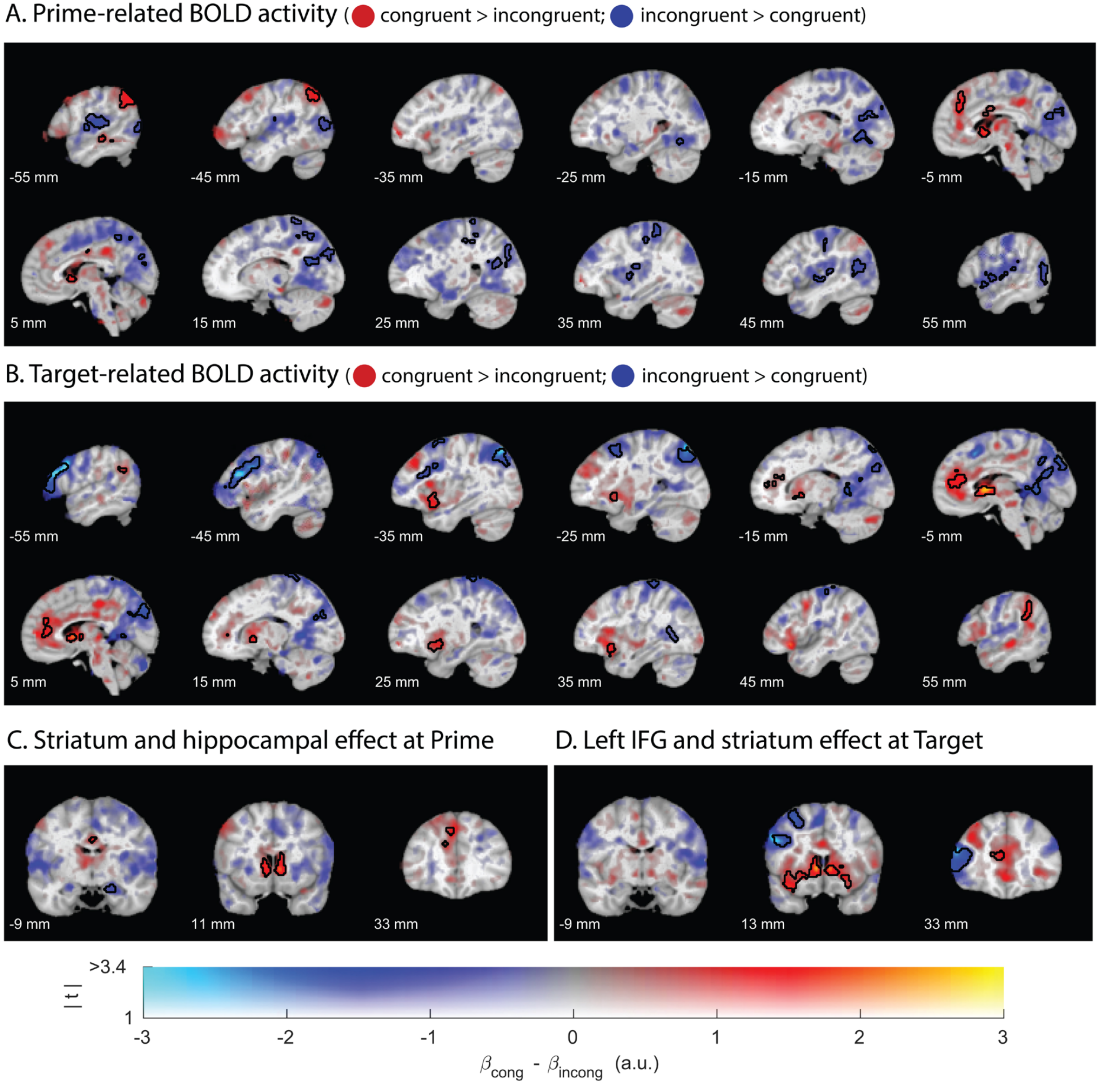
Univariate fMRI effects of novel meaning composition (prime-related activity) and its representational outcome (target-related activity). In red: order-congruent > order-incongruent; in blue: order-incongruent > order-congruent**.** (A) fMRI effects of order-congruent versus incongruent prime-related BOLD activity engages a broad temporoparietal network. (B) fMRI effects of order-congruent versus incongruent target-related BOLD activity (in blue: fMRI adaptation) reveal composed meaning representations in the left inferior cortex. (C) Prime-related effects of interest in the hippocampus and the striatum. (D) Target-related fMRI adaptation effects in the left IFG (in blue) but absent in the hippocampus. The hue indexes the sign and size of the contrast parameter estimate (congruent minus incongruent), and the opacity indexes the magnitude of the associated t values. Significant clusters (cluster-level corrected, FWE, *p* < .05) are encircled in solid contours. All coordinates are provided in the MNI space.

Comparison of fMRI BOLD responses during *primes* (i.e., when participants first encountered the novel pseudo-words) showed greater activity for order-incongruent than congruent primes in multiple temporal and parietal areas, including the precuneus, the postcentral gyrus, and the lingual gyrus (Fig. 2A, Supplementary Material 5A). Analysis of incongruent versus congruent *targets* revealed greater adaptation (and/or prediction error) of fMRI activity in a broad network of brain regions, including the middle frontal gyrus (Fig. 2B; Supplementary Material 5A) and the left inferior frontal cortex (Fig. 2D; *p*_FWE_ < .001, K_E_ = 1118, Z_max_ = 4.47, MNI coordinates of the peak = [-50, 33, 10], Supplementary Material 5B), a region often associated with deriving new and complex meaning from the lexical building blocks (Hagoort, 2005, 2016; Nevat et al., 2017; Weber et al., 2016; W. Zhang et al., 2022). Notably, the reverse contrast revealed greater activation during the congruent versus incongruent condition in the striatum, both at the time of the prime and the target (Fig. 2C, 2D; Supplementary Materials 5A).

Additional analyses within small volumes of interest revealed greater activity during incongruent than congruent primes in the hippocampal formation (Fig. 2C, *p*_FWE_ = .045, K_E_ = 26, Z_max_ = 3.72, [17, -9, -20]), perhaps reflecting greater effort to resolve the generalization-based composition challenge during incongruent than congruent primes. There was no evidence for effects of prime type in the mPFC (no suprathreshold clusters found after small volume correction, SVC). In contrast to our hypothesis, during target words there was no evidence for differences in fMRI adaptation in the hippocampal formation after congruent versus incongruent primes (Fig. 2D, *p*_FWE_ = .203, K_E_ = 6, Z_max_ = 3.50, [29, -38, 4], SVC). Moreover, the activity in mPFC was actually greater during targets following congruent than incongruent primes (Fig. 2B; *p*_FWE_ = < .001, K_E_ = 628, Z_max_ = 4.26, [-12, 41, 20]). Together, these results suggest that novel word meanings were represented in the middle frontal gyrus and left inferior frontal cortex, but not the predicted medial prefrontal-hippocampal network.

A supplementary analysis of contrasts between the congruent and uninterpretable conditions revealed qualitatively similar patterns of effects, thus substantiating these univariate analyses that focused on the congruent vs incongruent contrast (Supplementary Material 5C).

In sum, the process of composing meaning based on abstract relational structure rules, as measured in terms of prime-related BOLD signal, was associated with neural activity in a broad temporoparietal network, including the hippocampal formation. In addition, the representational outcome of this compositional process surfaced as fMRI adaptation of target-related neural activity in areas often associated with language processing, including the left inferior frontal cortex. Finally, successful meaning composition at target was accompanied by BOLD change in the striatum and the mPFC, perhaps reflecting intrinsic reward signaling.

### 3.3 Abstract rule representations in a lateral frontoparietal network

**Fig. 3.**
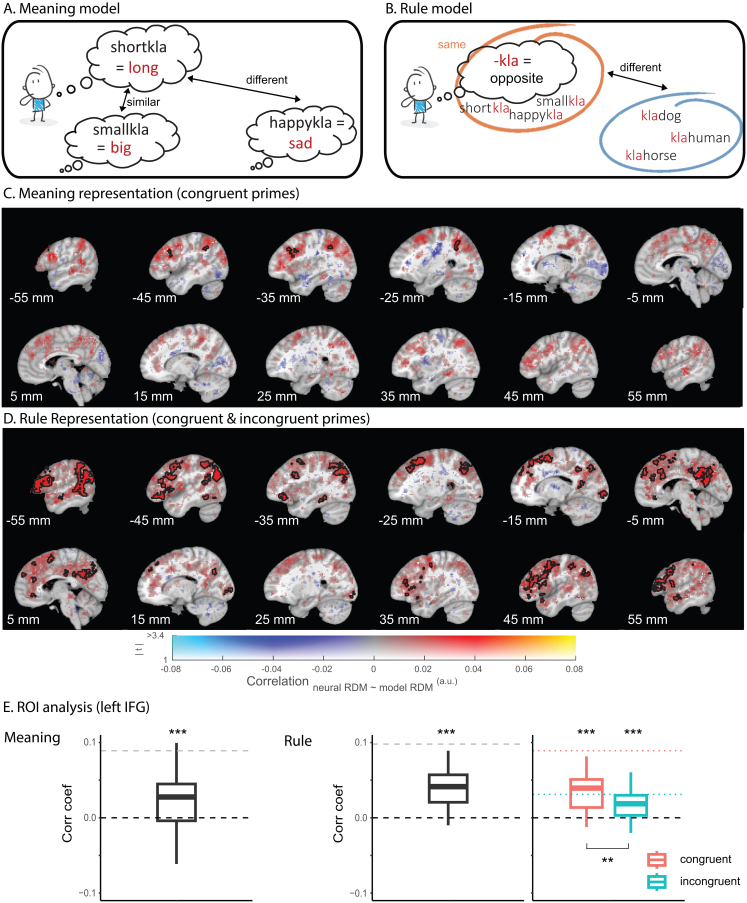
Representational similarity analysis of meaning and rule representations. (A) A distance-based meaning model in which word meanings are arranged by their similarities. (B) A binary-coded rule model in which all the compositional pseudo-words ending with the same affix (e.g., “-kla”) are more similar to each other, compared with words with a different affix (e.g., “kla-”, “-ran”, or “ran-”). (C) Whole-brain searchlight RSA outcome using the meaning model. Effects are shown from an analysis in which only order-congruent primes were included. (D) Whole-brain searchlight RSA outcome using the rule model. Effects are shown from an analysis in which congruent and incongruent conditions were combined. For both (C) and (D), the hue indexes the sign and size of the correlation coefficient, and the opacity indexes the magnitude of the associated t values. Significant clusters (cluster-level corrected, FWE, *p* < .05) are encircled in solid contours. All coordinates are provided in the MNI space. (E) ROI-based RSA of meaning and rule representations, extracted from the left inferior frontal gyrus. The analysis of meaning representations included only the congruent primes (left); whereas the analysis of rule representations included both congruent and incongruent conditions combined (middle) and separately (right). The ROI-based RSA was performed on both anatomically defined masks of the left IFG and the hippocampus. Hippocampal-based results is omitted in the figure due to low noise ceilings (see Supplementary Materials 6E). The gray horizontal line (or red/green line for the plot on the right) indicates the noise ceiling of the data extracted from the ROI, computed using a leave-one-out approach. The noise ceiling estimates the maximum performance any model could reasonably achieve given the noise in the data. Asterisks indicate the statistical significance: ***p < .001; **p < .01.

The fMRI adaptation effect at the time of the congruent vs incongruent *target* likely occurred because participants already composed the newly inferred word meaning using the abstract relational structure rules when they first encountered the primes. Indeed, RSA of congruent prime-related multivariate activity pattern using a whole-brain searchlight approach showed that the newly constructed meanings were represented in left lateralized language-related areas (Fig. 3C), including the left inferior frontal cortex (*p*_FWE_ = .001, K_E_ = 109, Z_max_ = 3.84, [-36, 25, 28]) and the angular gyrus (*p*_FWE_ < .001, K_E_ = 167, Z_max_ = 3.70, [-44, -46, 36], Supplementary Material 6B). The pattern of these RSA effects, at prime, overlapped greatly with the pattern of RSA effects, computed from target-related neural activity (predicted by the same target-meaning model; Supplementary Material 6C). Moreover, this left inferior frontal area overlapped greatly with the left frontal cluster yielded by the univariate fMRI adaptation analysis, which was the comparison between congruent vs incongruent targets. Importantly, this prime-related meaning representation was not captured by an alternative meaning model which described the similarities between stem meanings (e.g., “short” in “short-kla” was more similar to “small” in “small-kla”, compared with “happy” in “happy-kla”; Kendall’s τ_stem-target_ = 0.13; all cluster-level *p*s > .541; Supplementary Material 6D). This confirmed that the decoded target meaning representation at the primes was not a result of the semantic relatedness between the target meaning and the stem meaning. Together, this RSA demonstrated that the representation of the novel meaning was already composed and decodable at the time of pseudo-word primes.

Whole-brain searchlight RSA of prime-related activity pattern (including both congruent and incongruent primes) revealed representations of the abstract rules in a bilateral frontoparietal network (Fig. 3D), including dorsolateral prefrontal cortex (*p*_FWE_ < .001, K_E_ = 1639, Z_max_ = 4.59, [59, 7, 14]), middle temporal gyrus (*p*_FWE_ < .001, K_E_ = 4619, Z_max_ = 5.48, [-54, -52, 0]), and medial prefrontal cortex (*p*_FWE_ < .001, K_E_ = 273, Z_max_ = 5.01, [-6, 25, 38], Supplementary Material 6B). These areas are commonly implicated in the representation of task-state spaces and abstract rules in working memory for goal-directed action planning (Cole, Reynolds, et al., 2013; Cole & Schneider, 2007; Dosenbach et al., 2007; Harding et al., 2015; Nee, 2021; Spreng et al., 2010; Vaidya & Badre, 2022; Zanto & Gazzaley, 2013).

In contrast to our hypothesis, there was no evidence for either meaning or rule representations in the hippocampal formation, also not when reducing the search volume to an anatomically defined ROI (Supplementary Material 6E).

To explore the potential difference in rule representations between the order-congruent and incongruent primes, we performed the same analysis on the two conditions separately. Interestingly, ROI-based RSA in the left IFG revealed that the rule representation was stronger for the congruent than the incongruent conditions (Mean_cong_ = 0.04, SD = 0.03; Mean_incong_ = 0.02, SD = 0.02; Paired t(29) = 3.00, *p* = .005, Fig. 3E). This was further supported by whole-brain searchlight RSA of the congruent and incongruent conditions (Supplementary materials 6F).

In sum, our results showed that the online generalization of abstract rules for composing novel word meaning engaged a broad temporoparietal network including the hippocampus, with the newly composed words being represented in language-specific regions. During compositional generalization, both the newly inferred word meanings and the abstract rules could be decoded from a lateral frontoparietal control network, instead of the predicted medial prefrontal-hippocampal network.

## 4 Discussion

Our brain learns and abstracts generalizable knowledge to make inference and adapt to novel situations (Al Roumi et al., 2019; Behrens et al., 2018; Dehaene et al., 2022; Frankland & Greene, 2020; Gärdenfors, 2004; Liu et al., 2019; Sablé-Meyer et al., 2022; Schwartenbeck et al., 2023). Here, we leveraged fMRI adaptation and representational similarity analyses in the context of a new experimental procedure to investigate the neural representations that support this ability to compose novel word meaning based on previously learned abstract relational structure rules. Using fMRI adaptation, we demonstrated that newly inferred meanings are represented in the left inferior frontal cortex (IFC), a key region for constructing linguistic meaning (Nevat et al., 2017; Weber et al., 2016; W. Zhang et al., 2022). While we interpret the reduced neural activity for order-congruent versus incongruent targets as neural adaptation (see also Wagner et al., 1997), it is also possible that this difference reflects prediction error in response to incongruent targets (see also Weber et al., 2016). Although our current paradigm cannot distinguish prediction error from neural adaptation (cf. Todorovic & de Lange, 2012), both interpretations support the conclusion that novel word meanings must have been composed “on the fly”. Furthermore, the RSA-derived finding that novel meaning representations were decodable from the IFC already at prime onset reinforces the conclusion that the IFC represents the composed meanings themselves rather than representing prediction error. The conclusion that these representations reflect the newly composed meaning rather than the components of the pseudo-words is supported by the finding that the neural patterns at prime onset cannot be explained by a model of the meaning of the pseudo-word stems. It is remarkable that the covert products of the compositional inference process were already represented at the time of the prime. This finding captures our generative capacity, and the role of the inferior frontal cortex in representing constructed meaning.

While we observed hippocampal activity during compositional inference – specifically when participants encountered incongruent words compared with congruent ones, potentially reflecting greater effort to resolve the composition challenge – we found no clear evidence that the composed meanings themselves were represented in the hippocampus or the mPFC. This finding contradicts our preregistered hypothesis, which predicted that relational structure-based inference and generalization would engage representations within the medial prefrontal-hippocampal network, consistent with findings from other cognitive domains such as vision, memory, and planning (Baram et al., 2021; Barron et al., 2013, 2020; Behrens et al., 2018; Bellmund et al., 2018; Garvert et al., 2023). One possibility is that the involvement of this network depends on the format of the generalizable, abstract rule representations used for composition. The medial prefrontal-hippocampal network is thought to maintain task knowledge in a flexible cognitive map, with these map-like representations supporting novel shortcuts or connections that are never experienced (Barron et al., 2020; Garvert et al., 2023; Jacobs et al., 2013; Morton et al., 2020; Park et al., 2021; Schuck et al., 2016; Schuck & Niv, 2019; Schwartenbeck et al., 2023). In hindsight, the abstract rules of our task are unlikely to be formatted in terms of such a relational map; instead, they are more likely to be formatted as propositional or production rules (e.g., “if ‘kla’ is affixed at the end of a word, then it reverses the meaning of the word”) (Vaidya & Badre, 2022).

To assess the neural locus of the abstract rule representations, we conducted representational similarity analyses. Results revealed rule representations in a lateral frontoparietal network, including the dorsolateral prefrontal cortex (DLPFC) and lateral parietal cortex. These regions have previously been associated with the learning and use of abstract task representations for cognitive control (Badre et al., 2010; Cole, Laurent, et al., 2013; A. Eichenbaum et al., 2020; Ito et al., 2022; Loose et al., 2017; Nee, 2021; Reverberi et al., 2012; Tomov et al., 2018; Woolgar et al., 2011). This finding concurs generally with recent findings from an imaging study in which participants generalized (value-based) information across contexts based on learned abstract relationships that could also be conceptualized as being formatted as production rules (“if in context A, then category A+ gives more rewards”, Vaidya et al., 2021). These abstract production rules were also found to be represented in a similar frontoparietal control network, with flexible switching between rules facilitating efficient cognitive control. It is worth noting that while the rule model was designed to capture abstract rule representations, it may also reflect morpheme-based similarity driven by visual or positional properties of the stimuli. Our design cannot fully disentangle these influences from rule-based effects. That said, such lower-level visual or form-based similarity is typically associated with regions like the primary visual cortex or the visual word form area, rather than higher-order areas such as the dorsolateral prefrontal cortex, where we observed our key effects. Therefore, while we cannot entirely exclude lower-level contributions, our findings mostly reflect higher-level, abstract rule representations beyond basic stimulus properties. Nevertheless, the implication of the DLPFC in abstract rule representation is a post-hoc observation that warrants direct investigation in future research.

Notably, the brain region identified as representing newly composed meanings, the left IFC, is anatomically close to the DLPFC, a core area of the aforementioned cognitive control network associated with structure rule abstraction and novel inference (Badre et al., 2010; Bartolo & Averbeck, 2021; Braver et al., 2002; Monti et al., 2007; Morton et al., 2020; Ramawat et al., 2022; Wallis et al., 2001). This might suggest a shared system for linguistic and nonlinguistic generalization, with the left hemisphere playing a more prominent role in facilitating communication within the language network (Fiebach et al., 2005; Hagoort, 2016). This interpretation is further supported by our findings of left-lateralized parietal involvement in abstract rule representations, such as the angular gyrus, a region known to be involved in compositional semantic processes (Boylan et al., 2015; Price et al., 2015; W. Zhang et al., 2022).

To further explore neural signals associated with the compositional process, we compared fMRI BOLD responses during order-congruent versus incongruent primes. The widespread increases in activity across temporal and parietal regions likely reflect the critical difference between these conditions. This difference could be driven by unsuccessful inference of meaning, increase demands for rule application, or difficulty with meaning generalization. However, given the design of our study, we cannot disentangle these possibilities, and future research will be needed to clarify the underlying mechanisms.

In addition, when comparing congruent versus incongruent meaning compositions (both at the primes and at the targets), we observed increased neural activity in the striatum, a region often implicated in reward processing (Knutson et al., 2000, 2001). This finding is surprising because we did not provide participants with reward feedback regarding the accuracy of their semantic judgments; they were not informed which words were congruent and therefore meaningful. This finding might suggest that the process of generating meaning itself may provide an intrinsic reward. This aligns with the idea that internal rewards can facilitate the learning of grammar and new word meanings (Bains et al., 2024; Nevat et al., 2017; Ripollés et al., 2014; Ullman, 2016).

The observed weaker rule representation in the incongruent compared to congruent primes may reflect a process of rule switching. When participants encountered difficulty in composing a novel meaning under the incongruent rule (e.g., interpreting “kla-short” as “the young version of short”), they might shift to applying the reverse, congruent rule, which leads to a more interpretable outcome. This internal rule switching/shifting could result in a less stable representation of the assigned (incongruent) rule in the neural signal.

In sum, we investigated the neural representations that support novel compositional meaning inference. We leveraged the fact that participants can infer novel, compositional meanings on the fly, based on previously learned abstract rules (Tamminen et al., 2015; Zheng, Petukhova, et al., 2024). By using a semi-artificial language with a fully controlled and simplified compositional rule, we were able to isolate and probe core cognitive mechanisms underlying linguistic composition and generalization—mechanisms that are otherwise difficult to disentangle in naturalistic contexts. Using fMRI, we demonstrated that abstract rule generalization for composing novel meaning recruits processes and rule representations in the frontoparietal control network—in contrast to the predicted medial prefrontal-hippocampal network—and that the covert mental products of the compositional process can be decoded from the frontal cortex at the time of composition. The obvious next question is whether this paradigm can be leveraged to unravel the temporal dynamics of meaning composition. Future studies could use MEG to explore, for example: (1) whether word meaning representation in the left IFC reflects the transition from the stem word to the composed target word; (2) whether the representation of the presented abstract relational structure rule shifts to its reversed-order rule when the latter results in successful composition.

## Supplementary Material

Supplementary Material

## Data Availability

All the research data (e.g., data files, analysis scripts) associated with the current paper are shared in the Donders repository (https://doi.org/10.34973/45z9-a743).
