# Supplementary material for: Abstract rule generalization for composing novel meaning recruits a frontoparietal control network
Source: Imaging Neurosci (Camb). 2025 Oct 29;3:IMAG.a.963. doi: 10.1162/IMAG.a.963 (PMC12573890; doi:10.1162/IMAG.a.963)
Supplement: Supplementary Material [file IMAG.a.963_supp.docx]

# **Supplementary Materials**

## **Supplementary Material 1: Experimental Stimuli**

In total, six affix meanings were selected, each with 10 exemplar words (5 for learning, 5 for testing). These six affix meanings were assigned to three affix forms as prefix and suffix, respectively. We made sure that this sequential order rule could not be explained by the word class of the stems by misaligning the transitions (e.g., “-kla” turns an adjective to an adjective, whereas “kla-” turns a noun into a noun, however the same rule does not hold for affix “ran”, where both affix positions turn a noun into an adjective). The meaning-form assignment was counterbalanced across participants. In this way, we ensured the fMRI effects were not confounded by visual input. We also made sure that the compositional words do not resemble existing Dutch words.

For the testing list, we made sure that, in this case, there was no possibility of inferring any meaning for the incongruent words using the sequential order rule. Given the powerful compositionality of language, it turned out to be impossible to ensure that all the pseudo-words from the uninterpretable condition were meaningless. Instead, we ensured that in those rare cases, the meaning did not correspond to the target word. Note that there were twice as many items in the uninterpretable condition than in the congruent and incongruent conditions, due to the counterbalancing of the affix positions (e.g., short-kla was an order-congruent word, kla-short was an order-incongruent word, but both ran-short and short-ran were words from the uninterpretable condition). For all pseudo-words in the training phase and all order-congruent words in the testing phase, we made sure that there is an existing Dutch synonym (e.g. long, which is not itself a complex word, to avoid a direct mapping of affix meanings). This synonym (e.g. long) was paired with all three types of pseudo-words (kla-short, short-kla, and ran-short/short-ran), which were presented as a series of prime–target pairs, while participants were scanned with fMRI.

**Stimuli for Learning**


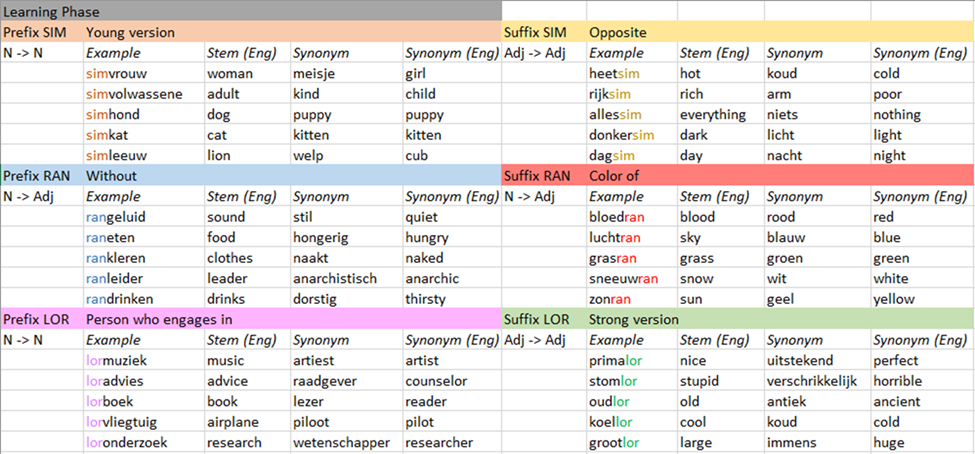


**Stimuli for Testing**


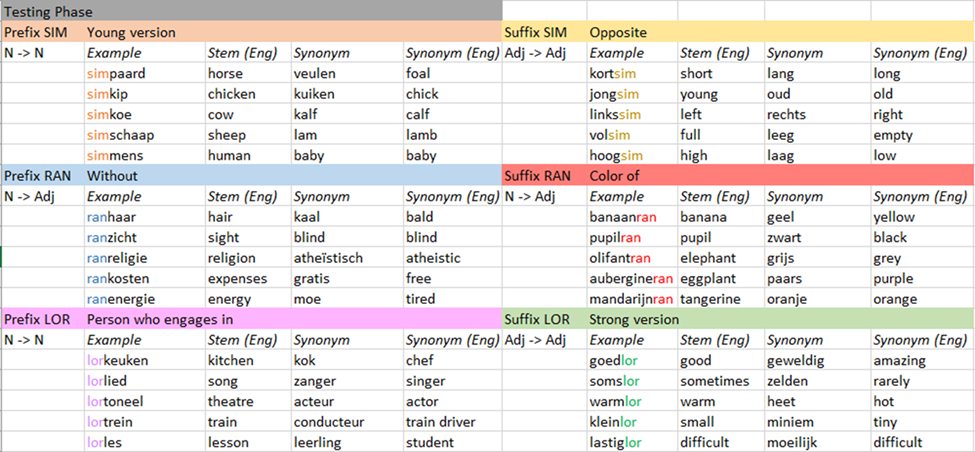


**Counterbalancing of affixations across participants in six lists.**

| **pre** | **post** | **pre** | **post** | **pre** | **post** |  |
| --- | --- | --- | --- | --- | --- | --- |
| **Young version** | **opposite** | **without** | **color of** | **person engaged** | **extreme version** |  |
| SIM | SIM | RAN | RAN | LOR | LOR | list1 |
| SIM | SIM | LOR | LOR | RAN | RAN | list2 |
| RAN | RAN | SIM | SIM | LOR | LOR | list3 |
| RAN | RAN | LOR | LOR | SIM | SIM | list4 |
| LOR | LOR | SIM | SIM | RAN | RAN | list5 |
| LOR | LOR | RAN | RAN | SIM | SIM | list6 |

## **Supplementary Material 2: MRI preprocessing**

The information below describes the anatomical and functional data preprocessing, which was retrieved directly from fMRIPrep.

**Anatomical Data Preprocessing.** The T1-weighted (T1w) image was corrected for intensity non-uniformity (INU) with N4BiasFieldCorrection (Tustison et al., 2010), distributed with ANTs 2.3.3 (Avants et al., 2008, RRID:SCR_004757), and used as T1w-reference throughout the workflow. The T1w-reference was then skull-stripped with a Nipype implementation of the antsBrainExtraction.sh workflow (from ANTs), using OASIS30ANTs as target template. Brain tissue segmentation of cerebrospinal fluid (CSF), white-matter (WM) and gray-matter (GM) was performed on the brain-extracted T1w using fast (FSL 6.0.5.1:57b01774, RRID:SCR_002823, Zhang et al., 2001). Brain surfaces were reconstructed using recon-all (FreeSurfer 6.0.1, RRID:SCR_001847, Dale et al., 1999), and the brain mask estimated previously was refined with a custom variation of the method to reconcile ANTs-derived and FreeSurfer-derived segmentations of the cortical gray-matter of Mindboggle (RRID:SCR_002438, Klein et al., 2017). Volume-based spatial normalization to one standard space (MNI152NLin2009cAsym) was performed through nonlinear registration with antsRegistration (ANTs 2.3.3), using brain-extracted versions of both T1w reference and the T1w template. The following template was selected for spatial normalization: ICBM 152 Nonlinear Asymmetrical template version 2009c (Fonov et al., 2009, RRID:SCR_008796; TemplateFlow ID: MNI152NLin2009cAsym).

**Functional Data Preprocessing.** For each of the 3 BOLD blocks per participant, the following preprocessing was performed. First, a reference volume and its skull-stripped version were generated by aligning and averaging 1 single-band references (SBRefs). Head-motion parameters with respect to the BOLD reference (transformation matrices, and six corresponding rotation and translation parameters) are estimated before any spatiotemporal filtering using mcflirt (FSL 6.0.5.1:57b01774, Jenkinson et al., 2002). BOLD blocks were slice-time corrected to 0.696s (0.5 of slice acquisition range 0s-1.39s) using 3dTshift from AFNI (Cox & Hyde, 1997, RRID:SCR_005927). The BOLD time-series (including slice-timing correction when applied) were resampled onto their original, native space by applying the transforms to correct for head-motion. These resampled BOLD time-series will be referred to as preprocessed BOLD in original space, or just preprocessed BOLD. The BOLD reference was then co-registered to the T1w reference using bbregister (FreeSurfer) which implements boundary-based registration (Greve & Fischl, 2009). Co-registration was configured with six degrees of freedom. First, a reference volume and its skull-stripped version were generated using a custom methodology of fMRIPrep. Several confounding time-series were calculated based on the preprocessed BOLD: framewise displacement (FD), DVARS and three region-wise global signals. FD was computed using two formulations following Power (absolute sum of relative motions, Power et al., 2014) and Jenkinson (relative root mean square displacement between affines, Jenkinson et al., 2002). FD and DVARS are calculated for each functional block, both using their implementations in Nipype (following the definitions by Power et al., 2014). The three global signals are extracted within the CSF, the WM, and the whole-brain masks. Additionally, a set of physiological regressors were extracted to allow for component-based noise correction (CompCor, Behzadi et al., 2007). Principal components are estimated after high-pass filtering the preprocessed BOLD time-series (using a discrete cosine filter with 128s cut-off) for the two CompCor variants: temporal (tCompCor) and anatomical (aCompCor). tCompCor components are then calculated from the top 2% variable voxels within the brain mask. For aCompCor, three probabilistic masks (CSF, WM and combined CSF+WM) are generated in anatomical space. The implementation differs from that of Behzadi et al. in that instead of eroding the masks by 2 pixels on BOLD space, the aCompCor masks are subtracted a mask of pixels that likely contain a volume fraction of GM. This mask is obtained by dilating a GM mask extracted from the FreeSurfer’s aseg segmentation, and it ensures components are not extracted from voxels containing a minimal fraction of GM. Finally, these masks are resampled into BOLD space and binarized by thresholding at 0.99 (as in the original implementation). Components are also calculated separately within the WM and CSF masks. For each CompCor decomposition, the k components with the largest singular values are retained, such that the retained components’ time series are sufficient to explain 50 percent of variance across the nuisance mask (CSF, WM, combined, or temporal). The remaining components are dropped from consideration. The head-motion estimates calculated in the correction step were also placed within the corresponding confounds file. The confound time series derived from head motion estimates and global signals were expanded with the inclusion of temporal derivatives and quadratic terms for each (Satterthwaite et al., 2013). Frames that exceeded a threshold of 0.5 mm FD or 1.5 standardised DVARS were annotated as motion outliers. The BOLD time-series were resampled into standard space, generating a preprocessed BOLD block in MNI152NLin2009cAsym space. First, a reference volume and its skull-stripped version were generated using a custom methodology of fMRIPrep. All resamplings can be performed with a single interpolation step by composing all the pertinent transformations (i.e. head-motion transform matrices, susceptibility distortion correction when available, and co-registrations to anatomical and output spaces). Gridded (volumetric) resamplings were performed using antsApplyTransforms (ANTs), configured with Lanczos interpolation to minimize the smoothing effects of other kernels (Lanczos, 1964). Non-gridded (surface) resamplings were performed using mri_vol2surf (FreeSurfer).

## **Supplementary Material 3: Functional and Anatomical ROIs**


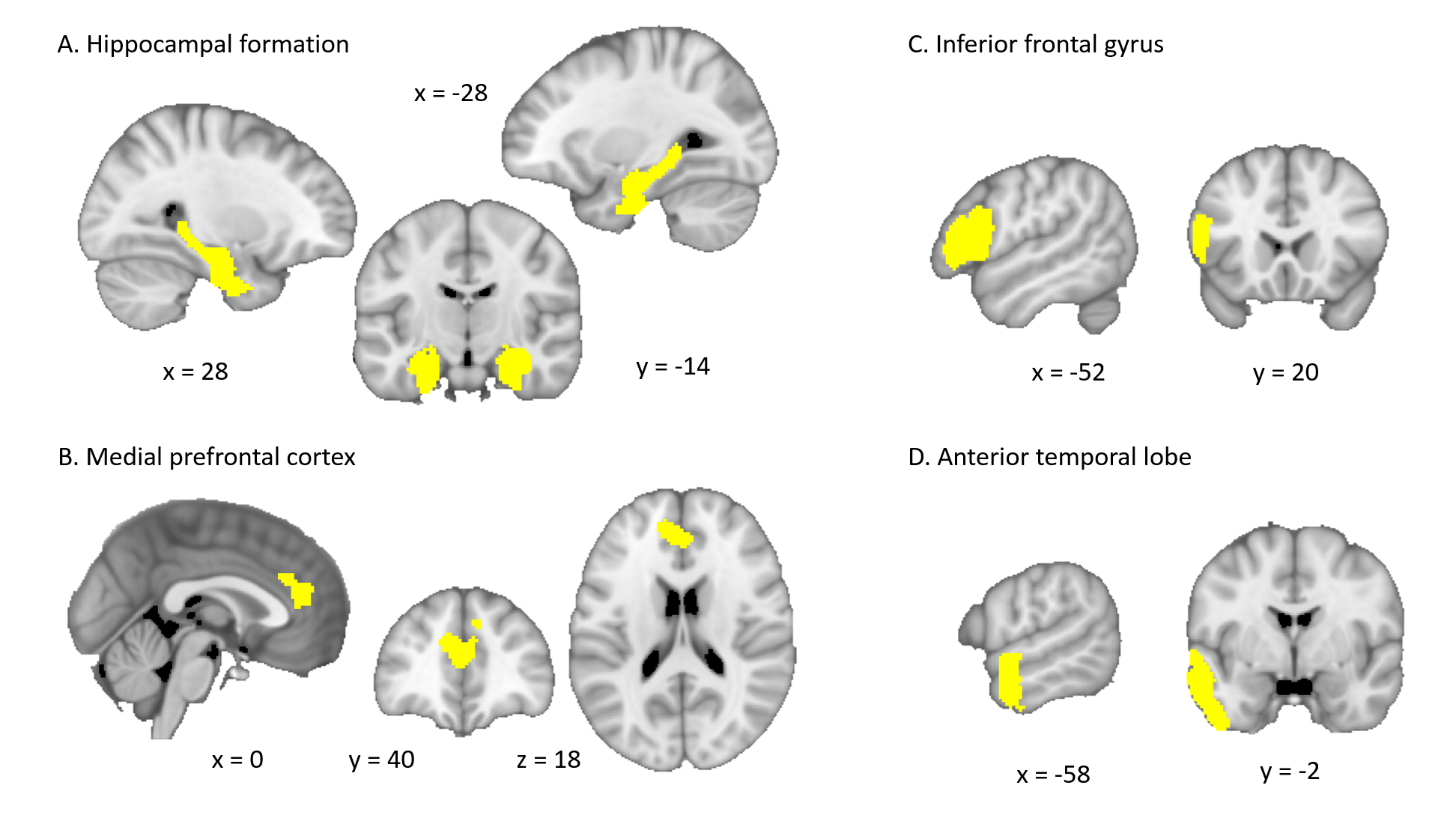


**Figure S3.** Functional and anatomical ROIs. (A) Anatomically defined ROI of the hippocampal formation, combining the hippocampal formation (incl. hippocampus, entorhinal cortex, subiculum). The hippocampal mask was defined using the Juelich histological atlas with a probabilistic threshold of 50%; (B) Functionally defined medial prefrontal (mPFC) ROI. The mPFC mask was defined based on previous study on conjunctive representation of building blocks in specific relational positions (t(29) > 2.5; Figure 3B in Schwartenbeck et al., 2023). (C) Anatomically defined ROI consisting of the left inferior gyrus (IFG). (D) Anatomically defined ROI consisting of the left anterior temporal lobe (ATL). Both (C) and (D) were defined using the Harvard-Oxford cortical structural atlas with a probabilistic threshold of 30%. All four masks were used for small volume correction (SVC) in the univariate analysis. Masks (A) and (B) were also used in the ROI-based multivariate RSA.

## **Supplementary Material 4: Full behavioral results**

**Learning.** During a pre-scanning training phase, 30 healthy participants were exposed to pairs of compositional pseudo-words along with their meanings (Figure 1A, see Supplementary Material 2 for a full set of stimuli). Each of these compositional pseudo-words comprises a known stem (e.g., “good” in “good-kla”) and an unknown affix (e.g., “kla”). We manipulated the mapping of the meaning to the affix based on their sequential position: e.g., “-kla” as a suffix means “the opposite”, whereas “kla-” as a prefix means “young version”. These position-dependent affixation rules allowed participants to compose unique meanings based on different sequential combinations of the affixes with the stems. Crucially, while the participants could infer the affixation rules from the exemplars, these rules were never made explicit to them. After four blocks of repetition, all participants were able to recall the meanings of the pseudo-words, evidenced by ceiling level performance on a subsequent memory task (mean_accuracy_ = 98.2 %, SD = 3.1%, Figure 1B).

**Testing (in the scanner).** To test participants’ knowledge of the abstract structure rules, we presented them with a new set of compositional pseudo-words they had never encountered before (e.g. “short-kla” and “kla-short”) while they were in the scanner, and asked them to imagine the meanings of the words. These novel words could be either congruent with the sequential order rule they had learned (e.g. for “short-kla”, “-kla” as suffix means “the opposite”, and the opposite of short is “long”), or incongruent (e.g. for “kla-short”, “kla-” as a prefix means “the young version of”, while it is much more difficult to infer the meaning of the young version of short). We employed an fMRI adaptation paradigm in which the pseudo-words (“primes”) were presented in pairs with their synonym (“targets”) matched to the congruent condition (e.g., “long” was presented after “short-kla” and also after “kla-short”, Figure 1C). After 10% of the targets, participants were presented a probe question about whether the meaning of the target word was the same as that of the preceding prime (i.e., the compositional pseudo-word). This manipulation allowed us to assess whether participants successfully inferred the meaning of the novel compositional words using the relational structure rules. Analysis of performance on these probe trials revealed significantly higher probability of meaning-match responses in congruent (mean = 90.7%, SD = 16.5%) than incongruent trials (mean = 23.8%, SD = 32.3%; β = 4.52, SE = 0.78, z = 5.80, *p* < .001; Figure 1D). This evidenced reliance on the relational structure rules for inference. In addition, we included a third group of pseudo-words primes where the stems were combined with alternative affixes, so that the meaning of the compositional pseudo-words were uninterpretable regardless of the position of the affix (e.g., ran-short = the color of short; short-ran = the person who engages in short). As a result, they also did not correspond to the target word meaning (e..g, ≠ long). Note that in most of the cases, these words in the uninterpretable condition were meaningless. As expected, participants did not consider these pseudo-words to match the meaning of the synonym (mean = 6.1%, SD = 7.8%). The degree to which the participants considered the novel word to match the synonym or not in the uninterpretable condition differed significantly from the congruent condition (β = 5.78, SE = 0.60, z = 9.60, *p* < .001), but not from the incongruent condition (β = 1.25, SE = 0.55, z = 2.27, *p* = .058; main effect between all three conditions: Χ^2^(2) = 93.51, *p* < .001).

**Posttest.** During the posttest, we asked participants explicitly to indicate whether they considered the novel pseudo-words that they had seen during the preceding MRI scan to be meaningful or not (Figure 1E). The pattern of the posttest results validated the probe results from the scanning session: The probability of “yes” responses to this question was significantly higher for the congruent pseudo-words than for the incongruent/uninterpretable pseudo-words (Figure 1F; mean_cong_ = 90.5%, SD_cong_ = 8.3%; mean_incong_ = 13.4%, SD_incong_ = 28.1%; mean_uninterpretable_ = 1.3%, SD_uninterpretable_ = 2.8%; Χ^2^(2) = 181.13, *p* < .001; congruent vs incongruent: β = 6.65, SE = 1.18, z = 5.64, *p* < .001; congruent vs. uninterpretable: β = 7.46, SE = 0.56, z = 13.43, *p* < .001; incongruent vs uninterpretable: β = 0.81, SE = 1.11, z = 0.73, *p* = .735).

At the end of the session, we also tested participants’ working memory capacity using an operation span task (Turner & Engle, 1989; Unsworth & Engle, 2005), where participants remembered letters while performing a mathematically calculation task (Mean = 49.3, SD = 12.7, range = 14-75). Additionally, we also collected information such as participants’ spoken foreign languages (Mean = 1.8, SD = 0.9, range = 1-4). This information were collected to explore the possible relationship between individual differences and learning of compositional rules (e.g., different strategies used, see Zheng, Petukhova, et al., 2024). However, we decided to skip the analysis given that participants did not show as much variance in their strategies as in the previous behavioral study.

## **Supplementary Material 5: Additional Univariate Analyses**

### ***Supplementary Material 5A: Full statistical outcome from the univariate fMRI analyses***

**Table S5A.** Univariate fMRI analysis of prime-related and target-related BOLD effects, as a function of congruent versus incongruent condition. Only significant clusters are included (*p* < .05, family-wise error corrected at the whole brain level). An uncorrected threshold of *p* < .001 is used for cluster forming. Results from the ROIs are reported in the main text.

| **location** | **voxels** | **p** | **z-max** | **Peak coordinates in MNI space** | **laterality** |
| --- | --- | --- | --- | --- | --- |
| **Prime-related effects (congruent < incongruent)** | | | | | |
| Cuneal cortex | 1007 | <.001 | 5.01 | [8, -76, 26] | bilateral |
| Planum temporale | 676 | <.001 | 5.05 | [43, -28, 10] | bilateral |
|  | 797 | <.001 | 4.73 | [-58, -19, 12] |  |
| Lateral occipital cortex | 377 | <.001 | 4.42 | [47, -62, 16] | bilateral |
|  | 203 | .001 | 4.55 | [-48, -84, 8] |  |
| Precuneus | 359 | <.001 | 4.32 | [11, -52, 56] | right |
| Postcentral gyrus/Precentral gyrus | 227 | <.001 | 4.52 | [37, -38, 62] | right |
|  | 106 | .035 | 4.12 | [27, -25, 48] |  |
| Lingual gyrus | 250 | <.001 | 4.19 | [-12, -62, -2] | bilateral* |
| Insular | 199 | .001 | 4.55 | [39, -5, 4] | right |
| **Prime-related effects (congruent > incongruent)** | | | | | |
| Striatum | 173 | .003 | 4.79 | [8, 11, 0] | bilateral |
|  | 323 | <.001 | 4.37 | [-6, 11, 0] |  |
| Angular gyrus | 483 | <.001 | 4.46 | [-56, -58, 42] | left |
| Middle temporal gyrus | 280 | <.001 | 4.33 | [-66, -44, -6] | left |
| Medial prefrontal cortex | 173 | .003 | 4.06 | [-12, 35, 28] | bilateral |
| **Target-related effects (congruent < incongruent)** | | | | | |
| Inferior frontal gyrus | 1118 | <.001 | 4.47 | [-50, 33, 10] | left |
| (Pre)cuneus | 1054 | <.001 | 4.46 | [2, -80, 36] | bilateral |
|  | 130 | .022 | 4.17 | [33, -60, 2] |  |
| Lateral occipital cortex | 531 | <.001 | 4.27 | [-28, -82, 44] | left |
| Postcentral gyrus | 333 | <.001 | 4.20 | [33, -36, 72] | right |
| Middle frontal gyrus | 296 | <.001 | 4.74 | [-30, 15, 56] | left |
| **Target-related effects (congruent > incongruent)** | | | | | |
| Striatum | 1106 | <.001 | 4.79 | [-6, 13, -2] | bilateral |
| Medial prefrontal cortex | 628 | <.001 | 4.26 | [-12, 41, 20] | bilateral |
| Angular gyrus | 293 | <.001 | 3.76 | [61, -40, 26] | bilateral |
|  | 112 | .042 | 3.93 | [-56, -50, 30] |  |

*denotes cases where clusters are present bilaterally in the uncorrected map, but only a cluster in one hemisphere survives correction.

### ***Supplementary Material 5B: Additional univariate analysis using small volume correction***

To probe the role of the language network in meaning inference, we additionally considered two anatomically defined masks, one for the left ATL and one for the left IFG). Apart from the above-mentioned fMRI adaptation effect in left IFG for incongruent versus congruent targets, there was no evidence for prime-related effect in the left IFG (no suprathreshold clusters found after SVC) nor target-related effect at left ATL (*p*_FWE_ = .082, K_E_ = 4, Z_max_ = 4.34, [-50, 1, -28], SVC). There was, however, greater activity in the left ATL during incongruent than congruent primes (*p*_FWE_ = .018, K_E_ = 22, Z_max_ = 3.71, [-62, -7, 2], SVC), which nevertheless merged with a bilateral temporal cluster and more likely reflected some general-purpose auditory processing (e.g., rehearsal of the composed meaning in order to make a comparison at target).

### ***Supplementary Material 5C: Univariate analysis of congruent versus uninterpretable contrasts***

**Prime-related Activities.** Comparison of congruent versus uninterpretable prime-related fMRI activity revealed less activity in a multiple temporal and parietal areas, including the precuneus, the lateral occipital cortex, and the lingual gyrus (Figure S5C, Table S5C). There was less activity during congruent than uninterpretable primes in the hippocampal formation (*p*_FWE_ = .003, K_E_ = 84, Z_max_ = 4.19, [19, -9, -18], SVC), perhaps reflecting less efforts to resolve the generalization-based composition challenge during congruent than uninterpretable primes. There was no evidence for any effect of prime type on activity in the mPFC or the left IFG (no suprathreshold clusters found after SVC). There was less activity in the left ATL during congruent than uninterpretable primes (*p*_FWE_ = .034, K_E_ = 14, Z_max_ = 3.50, [-60, -9, 2], SVC), but merging with a bilateral temporal cluster. Interestingly, we observe greater activation during congruent versus uninterpretable primes in the striatum.

**Target-related Activities.** Comparison of neural signals at the target word after a congruent versus uninterpretable compositional prime word revealed greater fMRI adaptation in a broad network of brain regions, including the (pre)cuneus, the postcentral gyrus, and the middle frontal gyrus (Figure S5C; Table S5C). Critically, we also observed greater adaptation in the left inferior frontal cortex. There was less activity in the left ATL during congruent than uninterpretable target (*p*_FWE_ = .003, K_E_ = 63, Z_max_ = 4.54, [-62, -5, 2], SVC), but again merging with a bilateral temporal cluster.

There was no evidence for fMRI adaptation in the hippocampal formation during target words after congruent versus uninterpretable primes (no suprathreshold clusters found after SVC). Moreover, also in contrast to our hypothesis, activity in mPFC was actually greater at targets following congruent than uninterpretable primes. In addition, a similar pattern of greater activation during congruent versus uninterpretable targets was seen in the striatum.


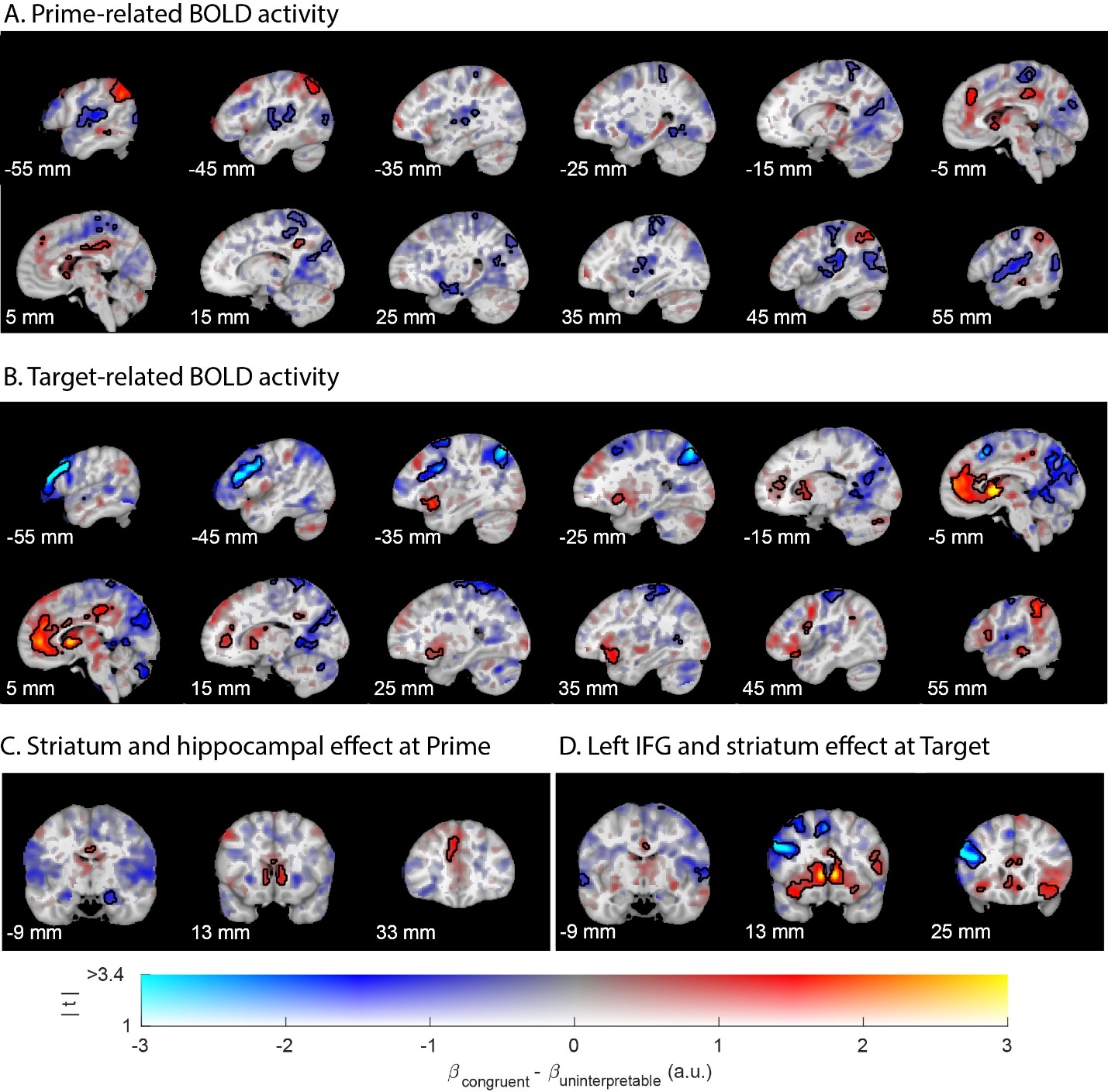


**Figure S5C.** Univariate fMRI effects of novel compositional words meaning computation (prime-related activity) and representation (target-related activity)**.** (A) fMRI effects of congruent versus uninterpretable prime-related BOLD activity. (B) fMRI effects of congruent versus uninterpretable target-related BOLD activity (in blue: fMRI adaptation). (C) Prime-related effects of interest. (D) Target-related effects of interest. The hue indexes the size of the parameter estimate, and the opacity indexes the unthresholded t values. Significant clusters (cluster-level corrected, FWE, *p* < .05) are encircled in solid contours. All coordinates are provided in the MNI space.

**Table S5C.** Univariate fMRI analysis of prime-related and target-related BOLD effects, as a function of congruent versus uninterpretable condition. Only significant clusters are included (*p* < .05, family-wise error corrected at the whole brain level). An uncorrected threshold of *p* < .001 is used for cluster forming. Results from the ROIs are reported below.

| **location** | **voxels** | **p** | **z-max** | **Peak coordinates in MNI space** | **laterality** |
| --- | --- | --- | --- | --- | --- |
| **Prime-related effects (congruent < uninterpretable)** | | | | | |
| Planum temporale | 2034 | <.001 | 5.34 | [63, -23, 8] | bilateral |
|  | 1460 | <.001 | 5.09 | [-60, -13, 6] |  |
| Lateral occipital cortex | 521 | <.001 | 5.03 | [51, -66, 18] | bilateral* |
| Amygdala | 206 | .001 | 4.78 | [33, -1, -24] | right |
| Temporal occipital fusiform cortex / lingua gyrus | 191 | .002 | 4.55 | [-28, -52, -10] | left |
| Precuneus | 239 | <.001 | 4.52 | [-16, -68, 18] | bilateral |
|  | 1523 | <.001 | 4.20 | [8, -52, 58] |  |
| Lateral occipital cortex | 158 | .006 | 4.29 | [-50, -82, 8] | bilateral |
|  | 521 | <.001 | 4.17 | [23, -78, 40] |  |
| **Prime-related effects (congruent > uninterpretable)** | | | | | |
| Striatum | 188 | .002 | 5.32 | [8, 13, 2] | bilateral |
|  | 132 | .016 | 4.51 | [-8, 9, 0] |  |
| Angular gyrus | 844 | <.001 | 5.24 | [-52, -56, 42] | bilateral |
|  | 347 | <.001 | 4.73 | [51, -48, 46] |  |
| Medial prefrontal cortex | 324 | <.001 | 4.69 | [-8, 37, 34] | bilateral |
| Posterior cingulate gyrus | 467 | <.001 | 4.97 | [-4, -34, 42] | bilateral |
| Middle temporal gyrus | 155 | .007 | 4.43 | [67, -23, -6] | bilateral |
|  | 230 | .001 | 4.32 | [-56, -40, -8] |  |
| **Target-related effects (congruent < uninterpretable)** | | | | | |
| Inferior frontal gyrus | 1561 | <.001 | 4.82 | [-42, 25, 26] | left |
| Lateral occipital cortex | 2919 | <.001 | 4.77 | [-32, -70, 44] | left |
| (Pre)cuneus |  |  |  | [2, -82, 30] | bilateral |
| Postcentral gyrus | 1396 | <.001 | 4.89 | [37, -36, 68] | right |
| Middle frontal gyrus | 386 | <.001 | 4.77 | [-34, 7, 64] | left |
| cerebellum | 294 | <.001 | 4.58 | [4, -82, -36] | right |
| Planum temporale | 257 | .001 | 4.42 | [65, -5, 4] | bilateral |
|  | 148 | .014 | 4.54 | [-62, -5, -2] |  |
| White matter | 143 | .017 | 4.27 | [33, -60, 4] | right |
| Paracingulate gyrus | 139 | .019 | 4.16 | [-4, 15, 52] | bilateral |
| **Target-related effects (congruent > uninterpretable)** | | | | | |
| Insular, Striatum | 5165 | <.001 | 5.67 | [-32, 17, -10] | bilateral |
| Medial prefrontal cortex |  |  | 5.63 | [6, 41, 8] | bilateral |
| Posterior cingulate gyrus | 435 | <.001 | 5.12 | [2, -32, 44] | bilateral |
| Angular gyrus | 318 | <.001 | 4.17 | [55, -46, 44] | right |
| Middle temporal gyrus | 243 | .001 | 5.03 | [61, -25, -14] | right |
| Inferior frontal gyrus | 182 | .005 | 4.14 | [49, 11, 26] | right |
| Cerebellum | 146 | .015 | 4.37 | [-20, -82, -34] | left |

*denotes cases where clusters are present bilaterally in the uncorrected map, but only a cluster in one hemisphere survives correction.

## **Supplementary Material 6: Additional RSA**

### ***Supplementary Material 6A: Visual RDM, Target-related activity***

Before conducting the RSA of interest, we first validated our analysis approach by using two visual model RDMs to predict the target-related neural activity. These RDMs reflect the visual similarity of the target words participants saw on the screen. The RDMs were computed as (1) the Levenshtein distance calculated using the “stringdist” library (van der Loo, 2014) in R; and (2) the pixel-wise Euclidean distance between individual words presented on the screen. As expected, the two RDMs were highly correlated (Kendall’s τ = 0.57, *p* < .001). RSA results showed that the visual similarity between target words was reflected in visual cortical areas. Thus, this control analysis confirmed our analysis approach.


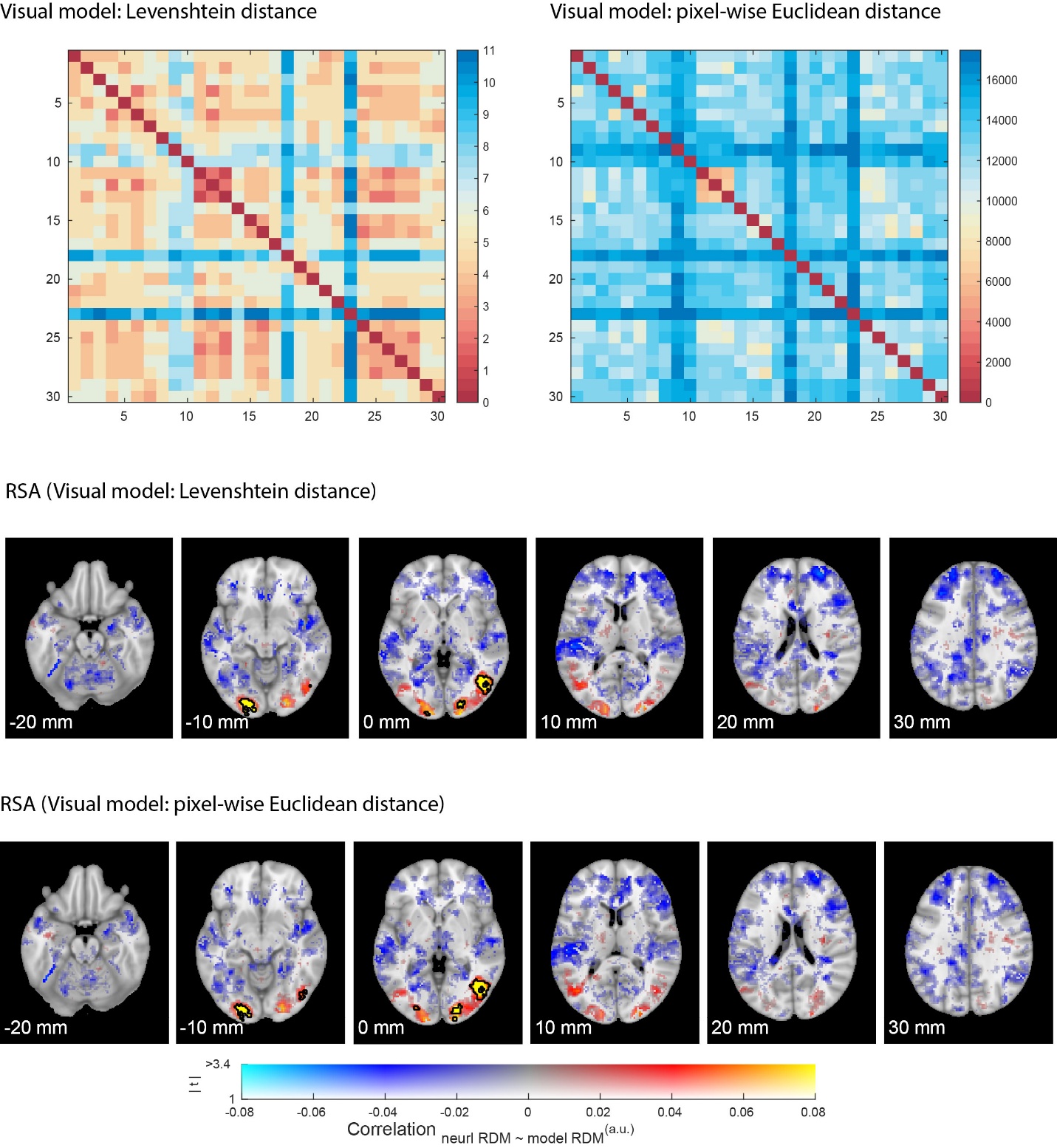


### ***Supplementary Material 6B: Full statistical outcome from the multivariate fMRI analyses***

**Table S6B**. Multivariate fMRI representational similarity analysis of prime-related BOLD signals, compared with the meaning model and the rule model, respectively. Only significant clusters are included (*p* < .05, family-wise error corrected at the whole brain level). An uncorrected threshold of *p* < .001 is used for cluster forming.

| **location** | **voxels** | **p** | **z-max** | **Peak coordinates in MNI space** | **laterality** |
| --- | --- | --- | --- | --- | --- |
| **Meaning model (congruent only)** | | | | |  |
| Inferior frontal gyrus | 109 | .001 | 3.84 | [-36, 25, 28] | left |
| Angular gyrus | 167 | <.001 | 3.70 | [-44, -46, 36] | left |
| **Rule model (congruent + incongruent)** | | | | | |
| Frontal pole/frontal orbital cortex | 3382 | <.001 | 5.52 | [-36, 31, -14] | bilateral |
|  | 201 | <.001 | 5.18 | [-8, 53, 34] |  |
|  | 90 | .001 | 4.24 | [31, 33, -8] |  |
| Middle temporal gyrus | 4619 | <.001 | 5.48 | [-54, -52, 0] | bilateral* |
|  | 216 | <.001 | 5.43 | [-60, -11, -10] |  |
| Inferior frontal gyrus | 1639 | <.001 | 4.59 | [59, 7, 14] | bilateral |
| Medial prefrontal cortex | 273 | <.001 | 5.01 | [-6, 25, 38] | bilateral |
|  | 59 | .014 | 4.06 | [-6, 35, -10] |  |
| Occipital pole | 168 | <.001 | 4.44 | [19, -94, -8] | bilateral |
|  | 135 | <.001 | 5.17 | [-18, -96, -8] |  |
| Lateral occipital cortex | 135 | <.001 | 4.28 | [49, -68, 30] | bilateral |
| Supramarginal gyrus | 117 | <.001 | 4.02 | [47, -40, 48] | bilateral |
| Superior temporal gyrus | 86 | .001 | 4.36 | [59, 1, -12] | bilateral* |
| Insula | 79 | .002 | 4.31 | [43, 21, -10] | right |
|  | 47 | .044 | 4.09 | [47, 15, -12] |  |
| Temporal fusiform cortex | 73 | .004 | 4.15 | [-38, -34, -16] | left |

*denotes cases where clusters are present bilaterally in the uncorrected map, but only a cluster in one hemisphere survives correction.

### ***Supplementary Material 6C: Target meaning RDM, Target-related activity***

Target-related neural representation was predicted by the same target-meaning model, reflected in a broad, left-lateralized network (incl. left IFC, left angular gyrus and left ATL).


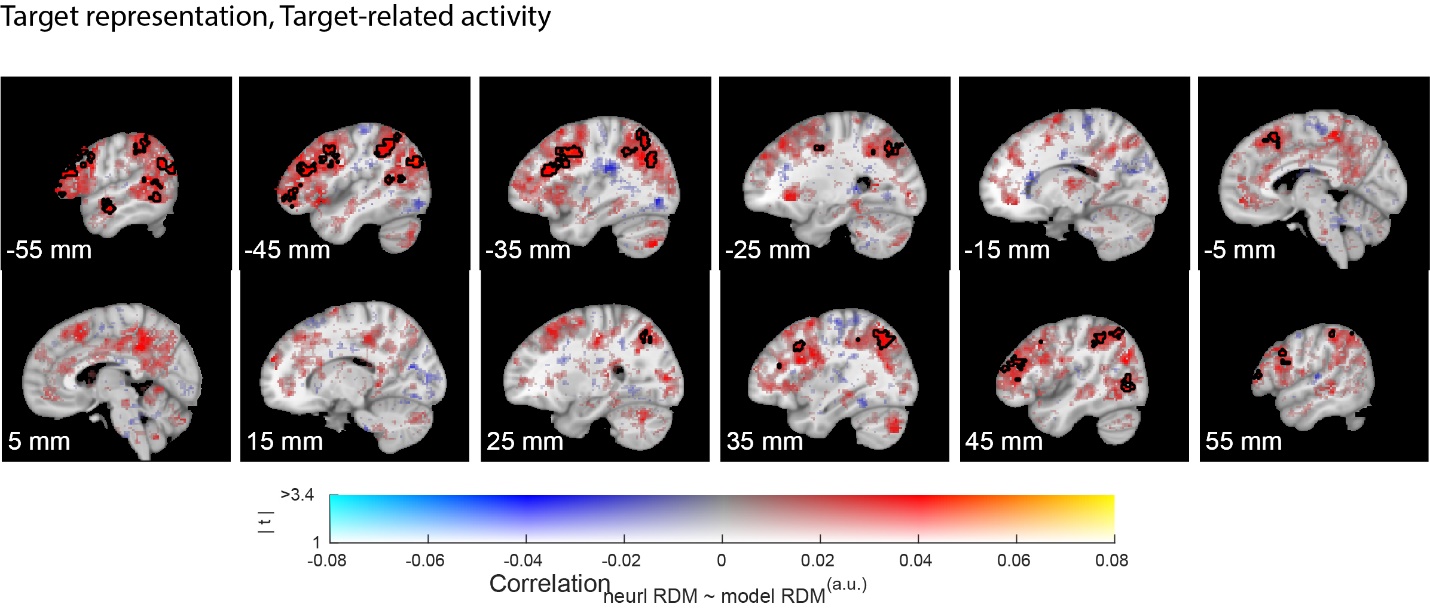


### ***Supplementary Material 6D: Stem meaning RDM, Prime-related activity***

Prime-related neural representation cannot be captured by an alternative meaning model which describes the similarities between stem meanings (e.g., “short” in “shortkla” is more similar to “small” in “smallkla”, compared to “happy” in “happykla”; Kendall’s τ_stem-target_ = .13; all cluster-level *p*s > .541).


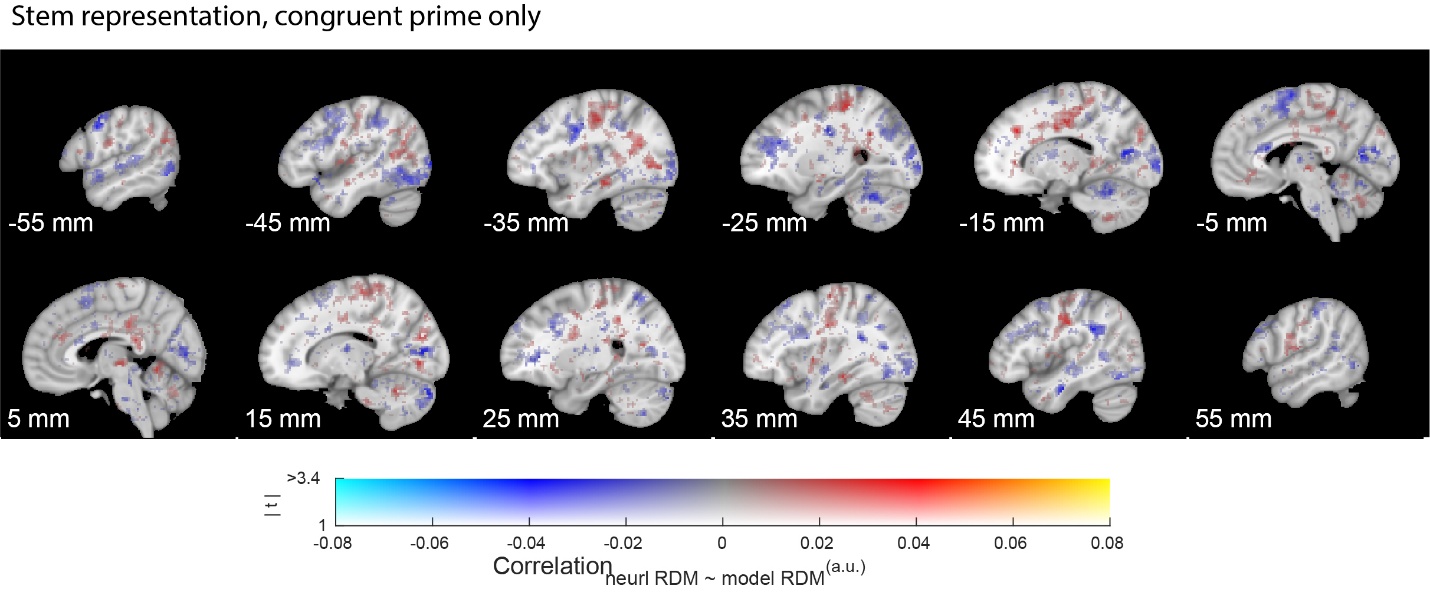


### ***Supplementary Material 6E******: Full ROI-based RSA***

Based on our a priori hypothesis and the univariate analysis, we further explored two ROIs: the hippocampus and the left IFG, using the masks also used in the univariate analysis. The ROI-based RSA (Figure 3E) confirmed the whole-brain results that left IFG represents both meanings (Mean_Kendall_τ_ = .03, SD = .05; t(29) = 5.11, *p* < .001) and rules (Mean_Kendall_τ_ = .04, SD = .03; t(29) = 7.70, *p* < .001). In contrast, the hippocampal results were unconvincing considering the very low noise ceiling (meaning representation: Mean_Kendall_τ_ = .002, SD = .03, t(29) = 0.57, *p* = 0.286, noise ceiling_cong_only_ = .006; rule representation: Mean_Kendall_τ_ = .01, SD = .03, t(29) = 1.45, *p* = .079; noise ceiling_congincong_combined_ = .004).

Furthermore, we explored the rule representation for congruent and incongruent primes respectively (Figure 3E, right panel). Our results showed that rule representation was better captured in the congruent condition compared with the incongruent condition (congruent: Mean_Kendall_τ_ = .04, SD = .03; t(29) = 6.44, *p* < .001; incongruent: Mean_Kendall_τ_ = .02, SD = .02; t(29) = 4.35, *p* < .001; Paired t(29) = 3.00, *p* = .005). The same pattern of differences between congruent and incongruent representations is confirmed in the whole-brain analysis (Supplementary Material 4E).

### ***Supplementary Material 6F: Full brain RSA of congruent and incongruent prime-related neural activities: rule representations***

Both the congruent and incongruent prime-related neural activities showed similar RSA outcome as the main analysis (i.e., when congruent and incongruent words were combined). The congruent condition showed a stronger left frontal and temporal effect than the incongruent condition in rule representation (Figure S6F).

**
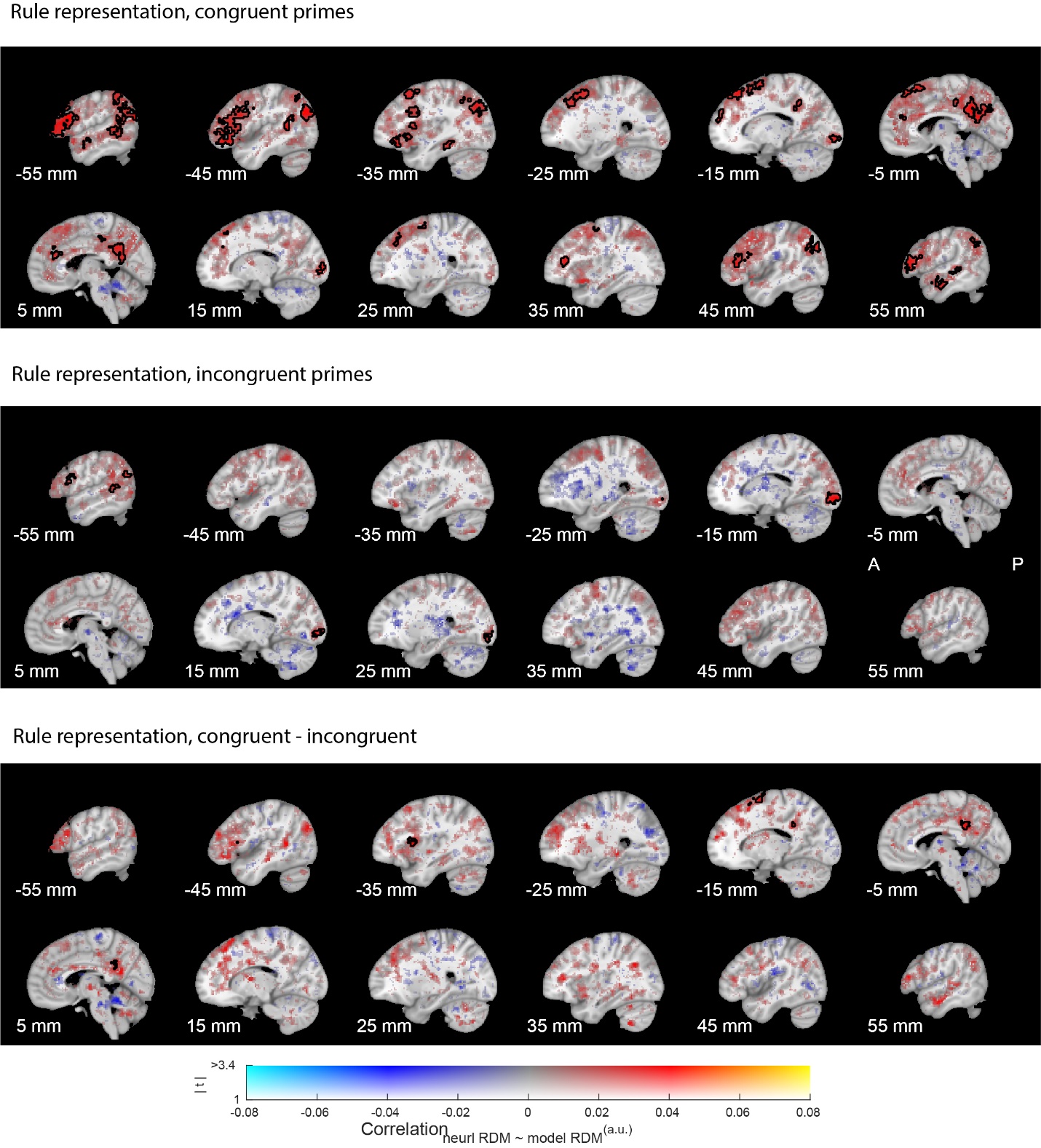
**

**Figure S6F.** Whole-brain searchlight RSA outcome using the rule model, performed for the congruent (top panel) and the incongruent (middle panel) conditions respectively, as well as their contrast (bottom panel).

## **References:**

Avants, B. B., Epstein, C. L., Grossman, M., & Gee, J. C. (2008). Symmetric diffeomorphic image registration with cross-correlation: evaluating automated labeling of elderly and neurodegenerative brain. *Medical Image Analysis*, *12*(1), 26–41. https://doi.org/10.1016/j.media.2007.06.004

Behzadi, Y., Restom, K., Liau, J., & Liu, T. T. (2007). A component based noise correction method (CompCor) for BOLD and perfusion based fMRI. *NeuroImage*, *37*(1), 90–101. https://doi.org/10.1016/j.neuroimage.2007.04.042

Cox, R. W., & Hyde, J. S. (1997). Software tools for analysis and visualization of fMRI data. *NMR in Biomedicine*, *10*(4–5), 171–178. https://doi.org/10.1002/(sici)1099-1492(199706/08)10:4/5<171::aid-nbm453>3.0.co;2-l

Dale, A. M., Fischl, B., & Sereno, M. I. (1999). Cortical surface-based analysis: I. Segmentation and surface reconstruction. *NeuroImage*, *9*(2), 179–194. https://doi.org/10.1006/nimg.1998.0395

Fonov, V., Evans, A., McKinstry, R., Almli, C., & Collins, D. (2009). Unbiased nonlinear average age-appropriate brain templates from birth to adulthood. *NeuroImage*, *47*(Supplement 1), S102. https://doi.org/10.1016/S1053-8119(09)70884-5

Greve, D. N., & Fischl, B. (2009). Accurate and robust brain image alignment using boundary-based registration. *NeuroImage*, *48*(1), 63–72. <https://doi.org/10.1016/j.neuroimage.2009.06.060>

Jenkinson, M., Bannister, P., Brady, M., & Smith, S. (2002). Improved Optimization for the Robust and Accurate Linear Registration and Motion Correction of Brain Images. *NeuroImage*, *17*(2), 825–841. https://doi.org/10.1006/nimg.2002.1132

Klein, A., Ghosh, S. S., Bao, F. S., Giard, J., Häme, Y., Stavsky, E., Lee, N., Rossa, B., Reuter, M., Neto, E. C., & Keshavan, A. (2017). Mindboggling morphometry of human brains. *PLoS Computational Biology*. https://doi.org/10.1371/journal.pcbi.1005350

Lanczos, C. (1964). Evaluation of Noisy Data. *Journal of the Society for Industrial and Applied Mathematics Series B Numerical Analysis*, *1*(1), 76–85. https://doi.org/10.1137/0701007.

Power, J. D., Mitra, A., Laumann, T. O., Snyder, A. Z., Schlaggar, B. L., & Petersen, S. E. (2014). Methods to detect, characterize, and remove motion artifact in resting state fMRI. *NeuroImage*, *84*, 320–341. https://doi.org/10.1016/j.neuroimage.2013.08.048

Satterthwaite, T. D., Elliott, M. A., Gerraty, R. T., Ruparel, K., Loughead, J., Calkins, M. E., Eickhoff, S. B., Hakonarson, H., Gur, R. C., Gur, R. E., & Wolf, D. H. (2013). An improved framework for confound regression and filtering for control of motion artifact in the preprocessing of resting-state functional connectivity data. *NeuroImage*, *64*, 240–256. https://doi.org/10.1016/j.neuroimage.2012.08.052

Turner, M. L., & Engle, R. W. (1989). Is working memory capacity task dependent? *Journal of Memory and Language*, *28*, 127–154. https://doi.org/10.1016/0749-596X(89)90040-5

Tustison, N. J., Avants, B. B., Cook, P. A., Zheng, Y., Egan, A., Yushkevich, P. A., & Gee, J. C. (2010). N4ITK: Improved N3 Bias Correction. *IEEE Transactions on Medical Imaging*, *29*(6), 1310–1320. https://doi.org/10.1109/TMI.2010.2046908.

Unsworth, N., & Engle, R. W. (2005). Individual differences in working memory capacity and learning: Evidence from the serial reaction time task. *Memory and Cognition*, *33*(2), 213–220. https://doi.org/10.3758/BF03195310

Zhang, Y., Brady, M., & Smith, S. (2001). Segmentation of brain MR images through a hidden Markov random field model and the expectation-maximization algorithm. *IEEE Transactions on Medical Imaging*, *20*(1), 45–57. https://doi.org/10.1109/42.906424.
